# NF-κB associated markers of prognosis in early and metastatic triple negative breast cancer

**DOI:** 10.1186/s13058-024-01925-3

**Published:** 2024-12-02

**Authors:** Payton De La Cruz, Julia McAdams, Melanie Morales Aquino, Aileen I. Fernandez, Andrew Elliott, Maryam Lustberg, Christoph Schorl, Jennifer R. Ribeiro, Nicole E. James

**Affiliations:** 1 Pathobiology Graduate Program, Brown University, Providence, Rhode Island USA; 2 Department of Obstetrics and Gynecology, Program in Women’s Oncology, Women and Infants Hospital, Providence, Rhode Island USA; 3 School of Public Health, Brown University, Providence, Rhode Island USA; 4 Caris Life Sciences, Inc., Phoenix, AZ USA; 5 Yale Cancer Center, Yale School of Medicine, New Haven, Connecticut USA; 6 Department of Molecular Biology, Cell Biology, and Biochemistry, Brown University Providence, Providence, Rhode Island USA; 7 Department of Obstetrics and Gynecology Warren-Alpert Medical School of Brown University, Providence, Rhode Island USA; 8 Department of Obstetrics and Gynecology, Program in Women’s Oncology, Women and Infants Hospital, 200 Chestnut Street, Room 208, Providence, Rhode Island 02903 USA

**Keywords:** Triple negative breast cancer, Immunotherapy, Immune checkpoint inhibitors, Biomarkers, NF-κB pathway

## Abstract

**Background:**

Triple negative breast cancer (TNBC) is the most aggressive subtype of breast cancer. While PD-1 based immunotherapies overall have led to improved treatment outcomes for this disease, a diverse response to frontline chemotherapy and immunotherapy still exist in TNBC, highlighting the need for more robust prognostic markers.

**Methods:**

Tumor-intrinsic immunotranscriptomics, serum cytokine profiling, and tumor burden studies were conducted in two syngeneic mouse models to assess differential effects in both the early-stage and metastatic setting. Bioinformatic analyses of both early and metastatic TNBC patient data were performed to assess if identified NF-κB-associated factors are associated with improved patient clinical outcomes.

**Results:**

NF-κB signaling driven by lymphotoxin beta expression is associated with tumor regression in TNBC mouse models. Furthermore, lymphotoxin beta expression in patient TNBC cohorts is prognostic of improved survival outcomes.

**Conclusions:**

This study highlights the potential role for NF-κB-associated factors, specifically lymphotoxin beta to be used as prognostic markers in TNBC, which could ultimately provide insight for improved targeted treatment approaches in the clinic.

**Supplementary Information:**

The online version contains supplementary material available at 10.1186/s13058-024-01925-3.

## Background

Triple negative breast cancer (TNBC) represents 15–20% of all breast cancer cases and exhibits higher rates of disease metastasis and recurrence rates, compared to all other subtypes [1]. Despite this relatively poor prognosis, TNBC is the only breast cancer subtype to benefit meaningfully from programmed-cell death protein 1 (PD-1)-based immunotherapy [2, 3]. In recent years, a plethora of clinical trials have demonstrated that PD-1-based immunotherapies improve treatment outcomes for TNBC patients [4–9], which quickly led to the incorporation of anti- PD-1 therapies with frontline, standard of care TNBC chemotherapeutic regimens. However, not all TNBC patients respond favorably to combinatorial immunotherapy and chemotherapy blockade [10–12]. This varied response to frontline treatment can be explained by the heterogenous nature of TNBC tumors, which are classified by a multitude of different molecular subtypes with diverse patient clinical outcomes [13, 14]. Interestingly, it has been established that different TNBC molecular subtypes exhibit distinct tumor immune microenvironment (TIME) features, such as varying levels of tumor infiltrating lymphocytes (TILs) [14, 15]. In addition, differences in early versus metastatic TNBC lesions are also known to exhibit different TIME characteristics [16]. Taken together, differential TNBC TIME dynamics can plausibly explain why some patients may represent more ideal immunotherapy candidates than others.

Current clinical guidelines recommend that intratumoral expression of PD-1’s ligand, programmed-death ligand 1 (PD-L1), be employed to determine whether PD-1-based immunotherapy should be incorporated in the frontline setting. However, its use remains controversial, as it is known that some patients with PD-L1 negative TNBC have favorable responses to PD-1 inhibition [16], while some PD-L1 positive TNBC tumors fail to derive benefit from anti-PD-1 immunotherapy. Interestingly, both the Impassion 031 and KEYNOTE 522 clinical trials, demonstrated a consistent benefit of combinatorial chemotherapy and PD-1 inhibition, irrespective of intratumoral PD-L1 expression in treatment naïve biopsies [17, 18]. Conversely, the GeparNuevo trial revealed that patients with PD-L1 positive tumors exhibited higher pathological complete response (pCR) rates regardless of if they were treated with chemotherapy alone or in combination with PD-L1 monoclonal antibody, duvalumab [19]. In addition, several studies in TNBC have reported that tumoral PD-L1 expression is associated with either poor prognosis, improved overall survival (OS), or no significant association with survival at all [8, 9]. Overall, the limited prognostic specificity of PD-L1 highlights the strong need for novel immune-based markers that can ultimately be implemented to provide insight on TNBC tumor heterogeneity and personalized treatment approaches.

In this current investigation, we sought to uncover novel intratumoral and circulating immunologic prognostic signatures using both early stage and metastatic immunocompetent TNBC in vivo models. These analyses will help to characterize how differential TIME dynamics impact patient prognosis, which can ultimately be leveraged to improve clinical outcomes.

## Methods

### Animals

Wild-type C57Bl/6 mice (strain #000664) and wild-type BALB/c mice (strain #000651) were obtained from Jackson Laboratories. All animal protocols were approved by the Brown University Animal Care and Use Committee (#22-09-0002) and were performed in accordance with the National Institutes of Health Guide for the Care and Use of Laboratory Animals. All animal protocols were reviewed and acknowledged by the Lifespan University Institutional Care and Use Committee (#1987412-1).

### Mouse studies and tissue collection

Mouse E0771 cells were obtained from American Type Culture Collection (ATCC) and cultured in DMEM, 10% FBS, and 1% penicillin/streptomycin. Cells were found to be free of pathogens and mycoplasma per Charles River pathogen testing. For the E0771 monotherapy study, eight-week-old female C57Bl/6J mice were injected with 100 µL of 5 × 10^5^ E0771 cell suspension in Matrigel or saline control into the 4th mammary pads under isoflurane sedation. Once palpable 14 days later, a group of pre-treatment mice were collected (*n* = 3), and remaining mice were randomly allocated into study groups to begin treatment with immune checkpoint inhibitor (ICI) monotherapy or control. Mice received 200 µg doses of mouse anti-PD-1 (clone: 29 F.1A12), anti-LAG-3 (clone: C9B7W), anti-TIM-3 (clone: RMT3-23), or rat IgG2a isotype control (clone: 2A3) every 4 days via intraperitoneal injection, with treatments stopping after the third dose. All antibodies used for in-vivo treatments were purchased from BioXcell. Doses were based on previously described tumor-reducing regimens [20]. One day after the final dose was administered, a cohort of “on-treatment” mice were collected and tumors were flash frozen and stored at -80 °C for further analysis. Remaining mice were monitored for 14 days and then collected as a “post-treatment” cohort. Tumors were obtained for all collections.

For the E0771 and 4T1 combination treatment studies, E0771 cells were cultured as described above, and mouse 4T1 cells were obtained from ATCC and cultured in DMEM, 10% FBS, and 1% penicillin/streptomycin. All cells were found to be free of pathogens and mycoplasma per Charles River pathogen testing. Eight-week-old female mice were injected with 100 µL of cells suspended in Matrigel into the 4th mammary pads under isoflurane sedation. C57Bl/6J mice received 5 × 10^5^ E0771 cells (*n* = 20 mice), while BALB/c mice received 1 × 10^5^ 4T1 cells (*n* = 20 mice). After the development of tumors (study day 14), E0771 and 4T1 mice were allocated into four treatment groups to receive anti-PD-1 mAbs, Carboplatin (McKesson, 0703-4246-01) and Paclitaxel (McKesson, 0703-3216-01) (referred to in text as Carbo/Pax), combinatorial treatment of anti-PD-1 with Carbo/Pax, or IgG isotype control. Mice receiving anti-PD-1 mAbs or IgG isotype control were injected with these therapies using the same dosing regimen as the E0771 ICI monotherapy study, while mice receiving Carbo/Pax were given 30 mg/kg of Carboplatin and 15 mg/kg Paclitaxel on study days 14 and 21. All mice were collected the day following the final treatments (study day 22), where serum and tumors were obtained. Whole blood was collected via cardiac puncture and serum was separated and stored at -80 °C as described above. Tumors were flash frozen and stored at -80 °C for further analysis. For both studies, tumor burden was determined by quantifying tumor weight as a proportion of total mouse weight.

### Tumor RNA isolation and NanoString nCounter® PanCancer IO360

RNA extraction from flash frozen on-treatment tumors (*n* = 3 per group) was performed using the Quick-RNA MiniPrep Kit (Zymo Research, R1054) with the manufacturer’s instructions. Briefly, tumors were homogenized in the supplied RNA lysis buffer before removing genomic DNA from the supernatant via spin column. RNA was precipitated from the flow-through with a 1:1 volume of 100% ethanol, then applied to RNA-binding columns. DNase treatment was performed on column membranes before being washed and eluted in nuclease-free water. RNA concentration and quality were quantified by the NanoDrop 2000 (Thermo Scientific, ND-2000) and subsequently stored at -80 °C. Gene expression levels were quantified via NanoString nCounter^®^ PanCancer IO360 mouse panel as previously described [21].

### NanoString nCounter® PanCancer IO360 analysis

Raw gene expression data was uploaded to the nSolver Advanced Analysis software package for quality control (QC) checks, background subtraction, and normalization. All samples passed automated QC checks. Normalization, fold changes, and p-values were obtained using criteria provided by NanoString (https://nanostring.com). Samples were grouped by treatment (*n* = 3 per group) for pairwise comparisons. Differential gene expression was reported with p-values and Benjamini-Hochberg false discovery rate adjusted p-values. Pathway and cell type profiling scores were determined as previously described [21]. Briefly, pathway scores for each sample were generated using Principal Component Analysis (PCA) on expression of genes associated with specific pathways, then scored based on expression of genes within the first PC. Cell type profiling scores, which approximate abundance of immune cell populations, were determined based on mRNA levels of cell type specific genes and adjusted using internal quality control markers. RCC files were deposited in NCBI’s Gene Expression Omnibus (GEO) and are accessible through GEO series accession number GSE279896.

### Protein extraction and western blots

On-treatment mouse tumors (15 mg per sample) were homogenized and sonicated on ice in lysis buffer with protease inhibitor, then agitated for 2 h at 4 degrees C. For E0771 and 4T1 cell lines, protein was extracted using Cell Lysis Buffer (Cell Signaling 9803) with 1 mM of protease inhibitor cocktail (AbCam, ab65621). All protein samples were then centrifuged at 13,000 RPM for 20 min at 4 degrees C. Total protein concentration was quantified from the supernatant via DC Protein Assay (Bio-Rad Laboratories, 5000116). Equal amounts of protein lysate from each sample were denatured and boiled at 70 degrees C for 10 min with Novex Sample Reducing Agent (Life Technologies, NP009) and NuPAGE LDS sample buffer (ThermoFisher Scientific, NP0007). Samples were electrophoresed through a 4–12% gradient SurPAGE™ Bis-Tris Gel (GeneScript, M000652). In a semi-dry transfer method, the Trans-Blot Turbo RTA Transfer Kit PVDF (Bio-Rad, 1704273) and Trans-Blot Turbo 5x Transfer Buffer (Bio-Rad, 10026938) were used to transfer the gel to a methanol-activated PVDF membrane in the Trans-Blot Turbo Transferring System (1.3 A, 25 V) for 10 min. Membranes were then blocked in 5% milk in phosphate-buffered saline with 0.05% Tween (PBST) for 20 min at room temperature. Primary antibodies were diluted in the 5% milk in PBST and incubated overnight at 4 degrees C. Membranes were washed 3x for 5 min in PBST, then incubated with the appropriate secondary antibodies diluted in 5% milk in PBST for 1 h at room temperature. Membranes were again washed 3x for 5 min in PBST before detecting HRP-linked secondary antibodies with Clarity™ Western ECL Substrate (Bio-Rad, Peroxide solution: 102030779; Luminol/enhancer solution: 102030787). The Bio-Rad ChemiDoc Imaging System was used to image all blots. Uncropped blots can be seen in Supplementary Fig. 1. Beta-actin was probed as a loading control. All antibodies and dilutions are as follows:

p50 (Cell Signaling, #13586 1:1000).

p52 (Cell Signaling, #4882, 1:1000).

PD-L1 (Proteintech, 66248-1-1 g, 1:500).

Beta-actin (Sigma, A5441,1:1000).

Anti-Rabbit (Cell Signaling, 7074 S, 1:1000).

Anti-Mouse (Cell Signaling, 7076 S, 1:1000).

### Serum cytokine multiplex assays

For all mouse studies, whole blood was obtained via cardiac puncture from post-mortem mice and collected into serum separator tubes. After clotting for 30 min, tubes were spun at 3000 g for 15 min at 4 °C. Serum supernatants were collected and stored at -80 °C. Serum was then analyzed in duplicate using the Mouse Cytokine/Chemokine 32-plex Discovery Assay Array (MD32) by Eve Technologies (Calgary, Canada) via Bio-Plex 200 bead analyzer. The following cytokines were quantified simultaneously in every sample: Eotaxin, G-CSF, GM-CSF, IFNγ, IL-1α, IL-1β, IL-2, IL-3, IL-4, IL-5, IL-6, IL-7, IL-9, IL-10, IL-12p40, IL-12p70, IL-13, IL-15, IL-17, IP-10, KC, LIF, LIX, MCP-1, M-CSF, MIG, MIP-1α, MIP-1β, MIP-2, RANTES, TNFα, VEGF-A.

### TCGA analysis

TNBC clinical outcome data and corresponding mRNA expression levels of genes of interest were obtained from the Breast Cancer METABRIC, Nature 2012 & Nat Commun 2016 study via cBioPortal [22, 23]. To obtain a TNBC patient cohort, the 3-gene classifier subtype was set to ER-/HER2-. Furthermore, “ER status” and “ER status by IHC” were set to negative. HER2 status was set to negative and HER2 status gain measured by SNP6 were removed from the analysis. Finally, upon downloading clinical outcome data any progesterone positive cases were removed leaving a total of 187 patient samples for analysis. All patients in this analysis had Stage I-III disease. For all OS analyses, patients coded as “died of other causes” were removed from analysis.

### Tumor immune dysfunction and exclusion

The Tumor Immune Dysfunction and Exclusion (TIDE) query gene function was performed to examine the Pearson correlation between *Ltb* and cytotoxic T lymphocyte (CTL) levels within the Breast METABRIC Triple-Negative cohort (*n* = 233) [24, 25].

### Real-world patient samples and outcomes

Retrospective analysis of 6102 cases with triple-negative breast carcinoma, including metastatic disease that were identified in a clinicogenomic database of de-identified solid tumors submitted to a Clinical Laboratory Improvement Amendments (CLIA)–certified laboratory (Caris Life Sciences, Phoenix, Arizona) for comprehensive genomic profiling. There were 6069 females and 33 males in the cohort, aged 19 to > 89 years, with a median age of 60 years. Next-generation sequencing of DNA (Illumina NextSeq, 592 genes, or Illumina NovaSeq, whole-exome sequencing) and RNA (Illumina NovaSeq, whole-transcriptome sequencing) were available for all cases, with genetic variant calling by board-certified molecular geneticists, as previously described [26]. RNA was isolated using a Qiagen RNeasy FFPE Kit (Germantown, MD); quality and quantity were determined using the Agilent TapeStation (Santa Clara, CA). Library preparation and whole-transcriptome sequencing were performed to an average of 60 M reads, as previously described [26].

Real-world OS and time on treatment (TOT) information were obtained from insurance claims data. OS was calculated from time of tissue collection to last contact. TOT was defined as the time from treatment initiation (pembrolizumab) to treatment discontinuation or last follow up. Patients were stratified based on the upper (> 75th percentile) and lower (< 25th percentile) quartiles of *Ltb* expression. Hazard ratio (HR) was calculated using the Cox proportional hazards model, and P values were calculated using the log-rank test.

### Tumor RNA isolation and quantitative PCR

RNA was isolated from tumors as described above (*n* = 3 per treatment group). Quantitative PCR was performed as previously described [27]. Validated mouse primers were purchased from Bio-Rad (*GAPDH*, *Ltb*, *Nfkb1*, *Nfkb2*, *Ifng*).

### Fluorescent immunohistochemistry

FFPE mouse tumors were stained for Ltb as described previously [27] and coverslipped in DAPI mounting medium (Vector Laboratories H-1200). Primary and secondary antibody and dilutions were as follows:

Ltb (OriGene, TA314161, 1:50).

Anti-Rabbit DyLight™488 (Vector Laboratories, DI-1488, 1:1,000.

### Image analysis

For both Ltb intensity, five randomly selected fields were selected based on DAPI staining. Images were acquired using a Zeiss Axio Imager M1 using diode lasers 402 and 488 using a 20x objective. Images were thresholded and mean intensity and integrated optical density (IOD) was calculated. Representative images were taken a 20x.

### Statistical analysis

For the E0771 monotherapy study, one-way ANOVA with post-hoc Tukey’s tests for multiple comparisons were performed to evaluate differences in tumor burden between treatment groups (*n* = 3 per group). Serum was analyzed for differences in cytokine levels between treatment groups using one-way ANOVA with post-hoc Tukey’s tests for multiple comparisons (*n* = 9 per treatment group). For the E0771 and 4T1 combination treatment study, one-way ANOVA with post-hoc Tukey’s tests for multiple comparisons were performed to evaluate differences in tumor burden between treatment groups of the same mouse model (*n* = 5 per treatment group). Unpaired Student’s t-tests were performed to compare tumor burden means between mouse models within the same treatment group. For serum cytokine levels within model groups, we performed one-way ANOVA with post-hoc Tukey tests to evaluate differences within models and to identify any differences in individual cytokines between treatment groups (*n* = 3 per treatment group).

## Results

### Enhanced NF-κB signaling and increased lymphotoxin beta expression are associated with anti-PD-1-mediated tumor regression in vivo

**Fig. 1:**
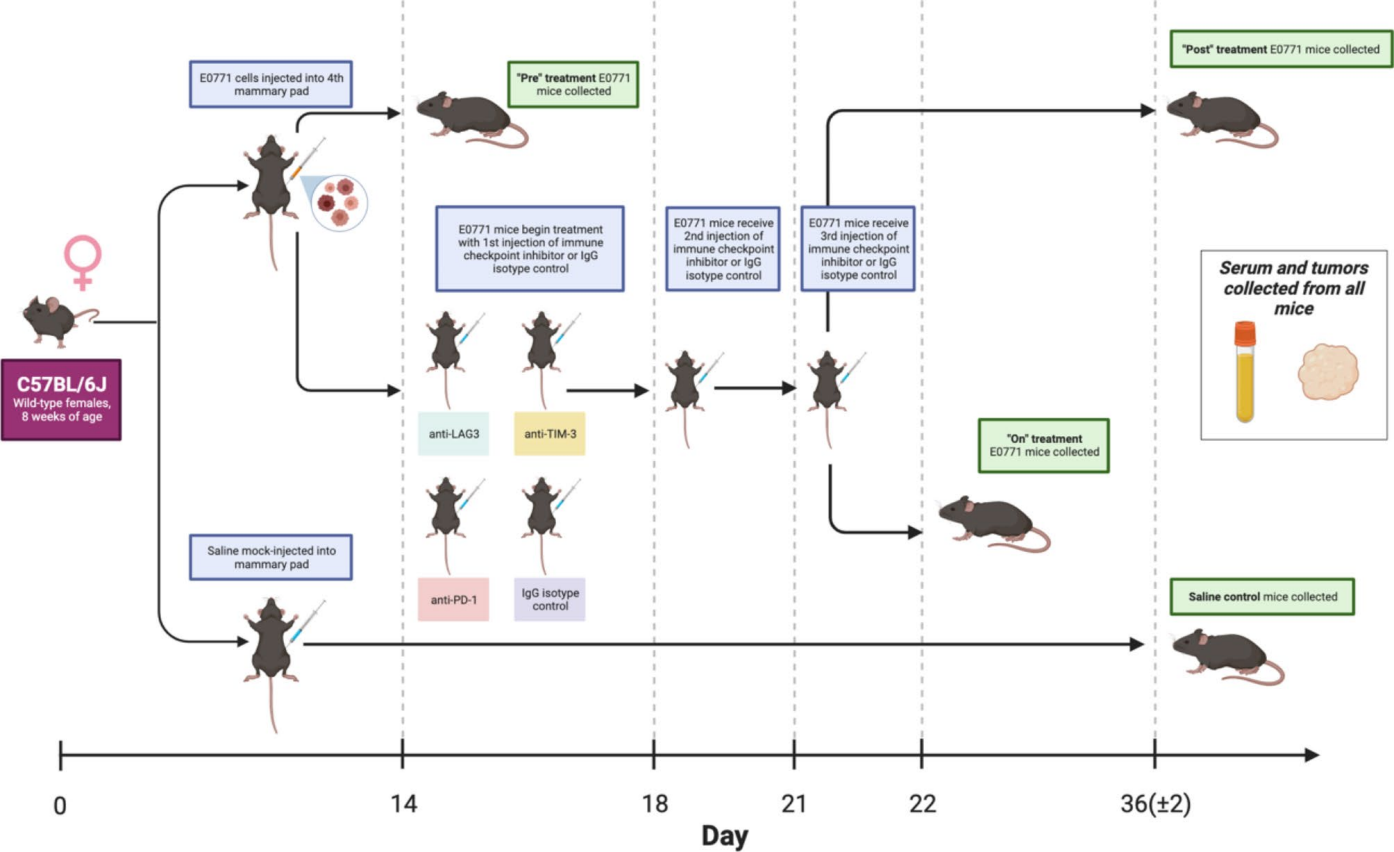
E0771 ICI monotherapy experimental timeline. Created with BioRender.com

To evaluate the effects of various immune checkpoint inhibitors on the tumor immune microenvironment, we performed an in vivo study in an immunocompetent mouse model using E0771 cells implanted into the mammary pad (Fig. 1). Tumor-bearing mice were divided into monotherapy treatment groups to receive 200 µg injections of anti-PD-1, anti-LAG-3, or anti-TIM-3 mAbs, or IgG isotype control. To assess temporal effects of ICI treatment, a cohort of mice was collected at an “on-treatment” timepoint 1 day following the third and final treatment, while another cohort was monitored and collected at a “post-treatment” timepoint 2 weeks later. At the post-treatment timepoint, anti-PD-1-treated mice showed almost complete tumor regression, which was statistically significant compared to the IgG isotype control group (*p* = 0.0125) (Supplementary Fig. 2). Mice treated with anti-LAG-3 or anti-TIM-3 exhibited a more variable response to treatment, with the anti-TIM-3-treated group achieving a significant reduction in tumor burden (*p* = 0.0492). Though the anti-LAG-3 group had a lower mean tumor burden than IgG isotype controls, this difference was not statistically significant (*p* = 0.8006).

**Fig. 2:**
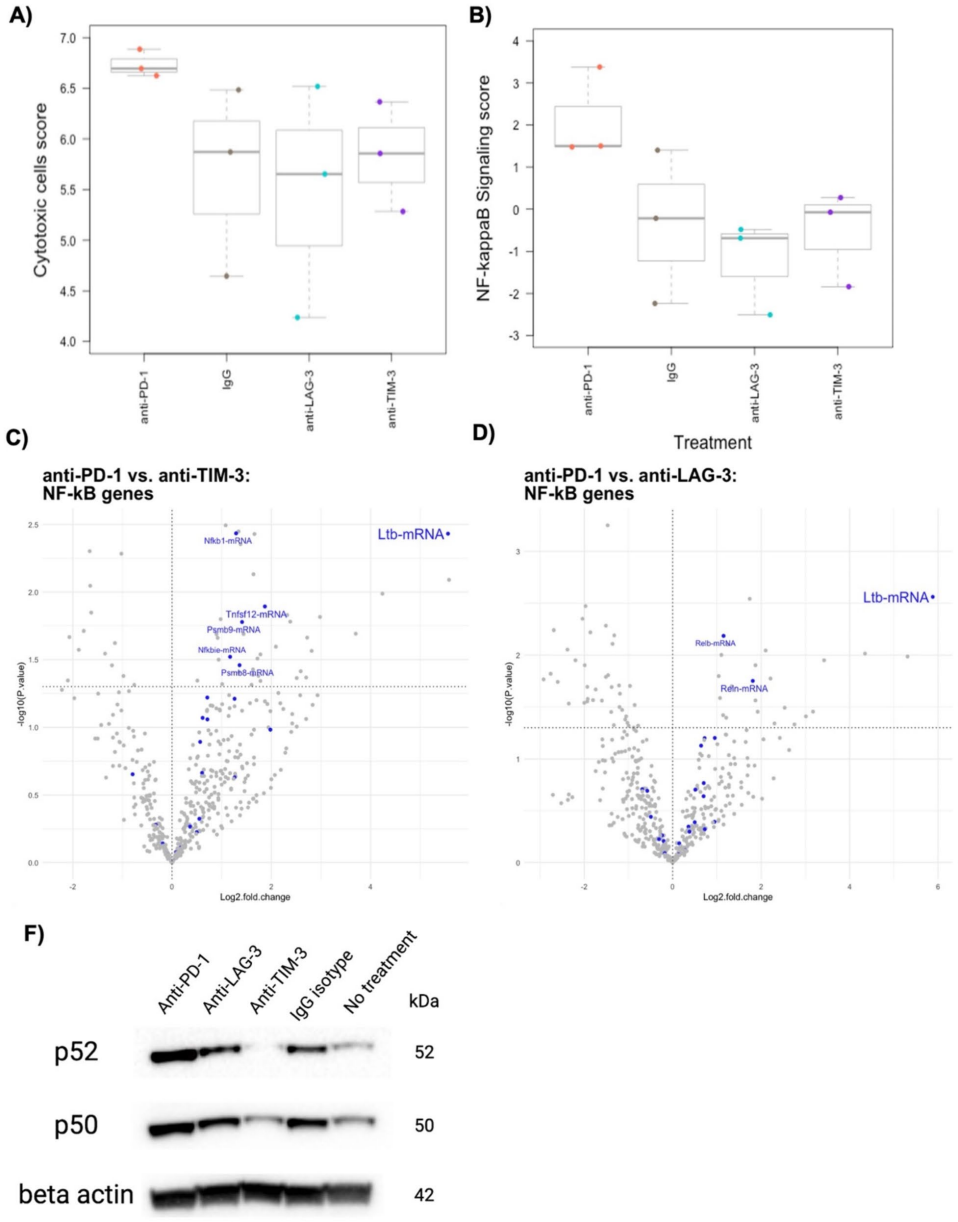
PD-1 inhibition is associated with enhanced NF-κB signaling in an E0771 TNBC in vivo model. On-treatment NanoString PanCancer IO360 cell type score analysis (*n* = 3 per group) demonstrates (**A**) increased infiltration of cytotoxic cells and (**B**) an elevated NF-κB signaling pathway scores in anti-PD-1 treated tumors. NF-κB signaling score differences were found to be driven by differential expression of Ltb when compared with the (**C**) anti-TIM-3 group and (**D**) anti-LAG-3 group. (**F**) Western blot analysis of on-treatment tumors shows increased levels of p52 and p50, markers for noncanonical and canonical NF-κB signaling, respectively. *p < 0.05, as indicated

To assess intratumoral factors associated with anti-PD-1-mediated tumor regression, NanoString Mouse PanCancer IO360 analysis was performed on tumors collected at the “on-treatment” timepoint. Pathway analysis revealed increased expression of genes associated with cytotoxic cells and NF-κB signaling in tumors from mice treated with anti-PD-1 mAbs (Fig. 2A-B) While no statistically significant differences were found, the anti-PD-1 group showed markedly higher scores for cytotoxic cells (Fig. 2A), specifically CD8 + T cells and NK cells. Taken together, these results may indicate a critical role for NF-κB signaling and NF-κB-specific mediators in potentiating cytotoxic activity and tumor regression in PD-1 blockade. All pathway and cell type score data can be found in Supplementary Fig. 3.

We then examined differential expression of genes in the NF-κB signaling pathway and found that *Ltb* was the top differentially expressed gene in the anti-PD-1 group (Fig. 2C-D). *Ltb* encodes for Lymphotoxin beta, a key upstream activator of NF-κB signaling that regulates lymphoid tissue development and is hypothesized to promote the development of ectopic lymphoid structures in the tumor microenvironment [28]. Treatment with anti-PD-1 also upregulated expression of *Relb* and *Reln* when compared to the anti-LAG-3 group, and *Nfkb1*, *Tnfsf12*, *Psmb9*, *Nfkbie*, and *Psmb8* when compared to the anti-TIM-3-treated group (Fig. 2C-D).

We then examined tumor-intrinsic levels of proteins associated with canonical and noncanonical NF-κB activation. The canonical and noncanonical NF-κB pathways involve different upstream regulators and downstream effectors, thereby playing distinct roles in immunity and disease. Ltb is known to activate the noncanonical NF-κB pathway via binding with Lymphotoxin beta receptor (LTBR), but a recent study by Legut et al. demonstrated that LTBR activation also promotes signaling through the canonical pathway [29]. Western blot analysis of on-treatment tumors showed that anti-PD-1 treated tumors indeed had high levels of p52 and p50, markers for noncanonical and canonical NF-κB activation, respectively, compared to controls (Fig. 2F). This finding suggests that both canonical and noncanonical NF-κB signaling is involved in response to anti-PD-1 therapy, and that there may be significant interplay between these two pathways.

### Treatment with anti-PD-1 immunotherapy elicits robust serum cytokine and chemokine profiles in vivo

**Fig. 3:**
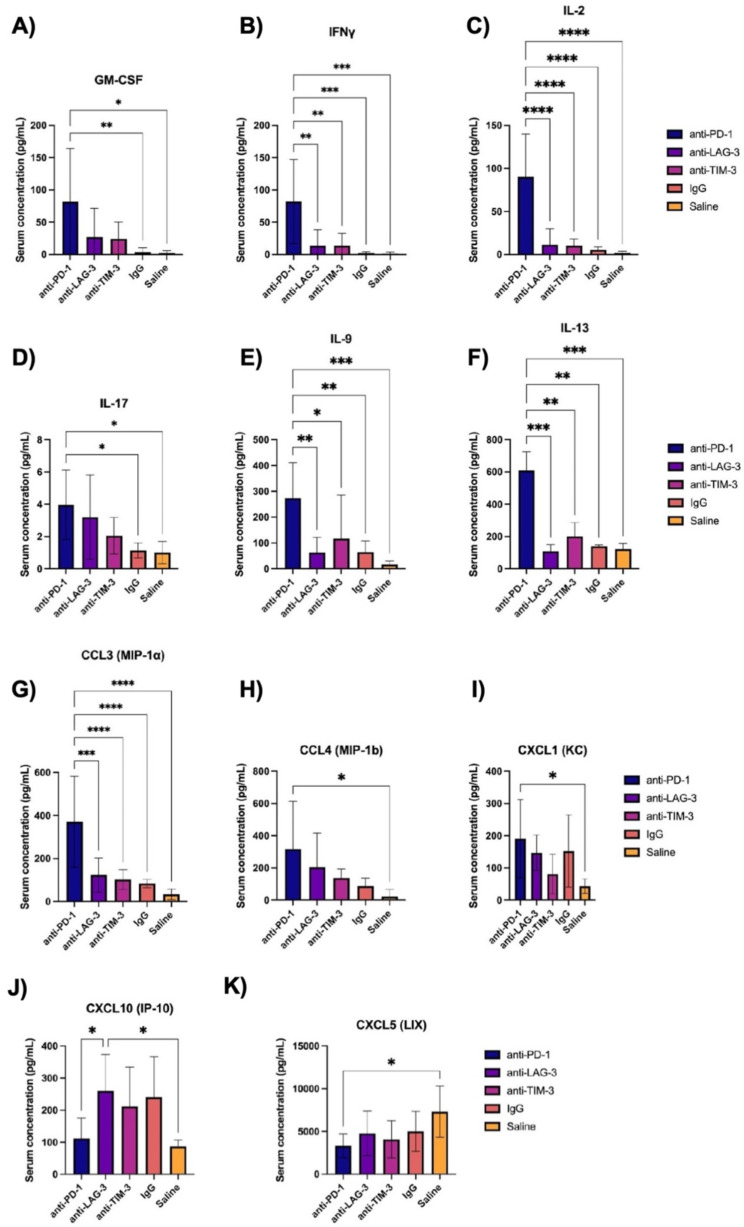
Serum cytokine analysis for E0771 ICI monotherapy study. Post-treatment serum levels of (**A**) GM-CSF, (**B**) IFNγ, (**C**) IL-2, (**D**) IL-17, (**E**) IL-9, (**F**) IL-13, (**G**) CCL3, (**H**), CCL4, (**I**) CXCL1, (**J**) CXCL10, and (**K**) CXCL5. *p < 0.05, **p < 0.01, ***p < 0.001, ****p < 0.0001, as indicated

Next, we aimed to uncover novel circulating immune signatures associated with immunotherapy response using a multiplex cytokine and chemokine array on serum obtained from post-treatment mice. One-way ANOVA with post-hoc Tukey tests for multiple comparisons were performed to evaluate differences in individual serum cytokines between treatment groups. PD-1 blockade elicited higher circulating levels of several proinflammatory cytokines when compared to IgG control, including GM-CSF (*p* = 0.01), IFNγ (*p* = 0.0003), IL-2 (*p* = 0.013), and IL-17 (*p* = 0.014) (Fig. 3A-D). GM-CSF, IFNγ, and IL-2 all have critical roles in stimulating cytotoxic immune cells and promoting antitumor activity, and are associated with beneficial responses to immunotherapy [30–32]. IL-17, a cytokine whose production is induced by NF-κB signaling, promotes the production of several other proinflammatory cytokines, such as IL-6 and IFNγ [33]. Anti-PD-1 treatment also significantly increased serum levels of pleiotropic cytokines, such as IL-9 (*p* = 0.0004) and IL-13 (*p* = 0.0014) (Fig. 3E-F). IL-9, produced by activated T cells, is known to inhibit tumor cell proliferation and promote cytotoxicity in solid tumors [34]. Interestingly, the role of IL-13, a key regulator in innate immunity, remains contested in cancer as it may exhibit either pro- or anti-tumorigenic effects depending on context [35]. Compared to all other treatment and control groups, anti-PD-1 therapy dramatically increased serum levels of the chemokine CCL3 (*p* < 0.0001), also known as MIP-1ɑ (Fig. 3G). CCL3, whose production is induced by NF-κB signaling, functions as a potent chemoattractant for T cells, macrophages, and other immune cells to the TIME [36]. Moreover, circulating levels of the NF-κB-regulated chemokines CCL4 (*p* = 0.0210) and CXCL1 (*p* = 0.0224), also known as MIP-1β and KC, respectively, were significantly elevated in the anti-PD-1 treatment group when compared to healthy controls who received mammary pad mock-injections of saline (Fig. 3.H-I). CCL4 has similar chemoattractant properties to CCL3 and has been suggested to promote antitumor immunity in response to immune checkpoint blockade [37]. Conversely, CXCL10, a pleiotropic chemokine also known as IP-10, was higher in serum of anti-LAG-3-treated mice when compared to anti-PD-1-treated mice and saline controls (*p* = 0.0466) (Fig. 3.J). In addition, circulating levels of CXCL5, also known as LIX, were significantly lower in the anti-PD-1 group compared to saline controls (*p* = 0.0237) (Fig. 3.K). CXCL1, CXCL5, and CXCL10 have generally been considered to be pro-tumorigenic in several cancer types, as they are associated with poorer prognosis and an immunosuppressive microenvironment [38–40].

**Fig. 4:**
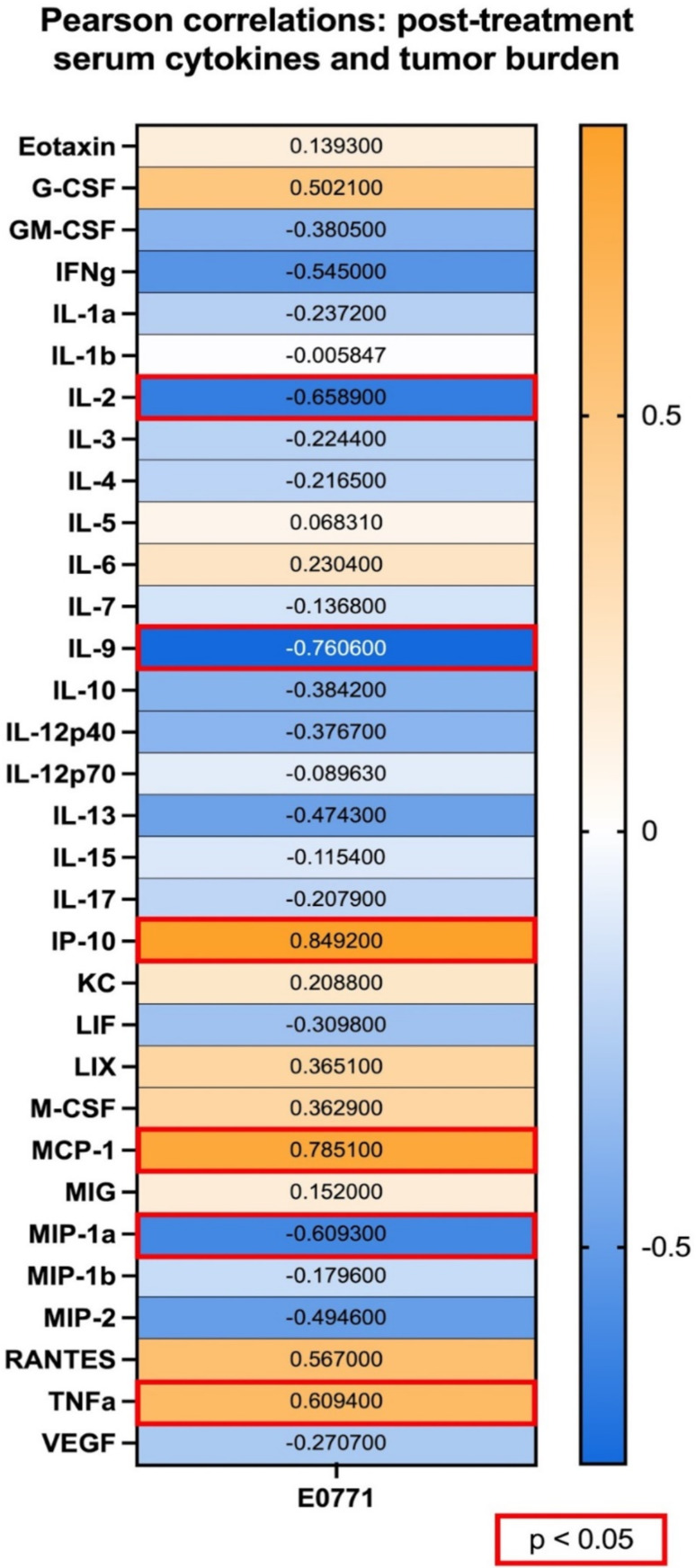
Pearson correlation analysis of post-treatment serum cytokine levels with tumor burden in E0771 ICI monotherapy study. *p < 0.05, as indicated

To evaluate potential relationships between individual cytokines and disease severity, regardless of treatment group, we calculated Pearson correlation coefficients for serum cytokine levels and tumor burden (Fig. 4). The cytokines that exhibited a particularly strong positive correlation with tumor burden were CXCL10 (IP-10) (*r* = 0.8492, *p* = 0.0009) and CCL2 (*r* = 0.7851, *p* = 0.0042), also known as MCP-1. CCL2 is a pro-tumorigenic cytokine that attracts TAMs and stimulates metastasis in breast cancer [41]. TNFɑ was also positively correlated with tumor burden (*r* = 0.6094, *p* = 0.0465), though not as strongly as CXCL10 or CCL2, indicating that higher serum levels of these cytokines may be associated with higher severity of disease. Conversely, strong negative correlations with tumor burden were found with IL-2 (*r*=-0.6589, *p* = 0.0275) and IL-9 (*r*=-0.7606, *p* = 0.0066). CCL3 (MIP-1ɑ) was also negatively correlated with tumor burden (*r*=-0.6093, *p* = 0.0466). Indeed, these results are consistent with our findings that IL-2, IL-9, and CCL3 are elevated in serum from anti-PD-1-treated mice, which may suggest that higher levels of these cytokines are indicative of PD-1 blockade response.

### Intratumoral Ltb expression predicts improved clinical outcomes in human TNBC patients

**Fig. 5:**
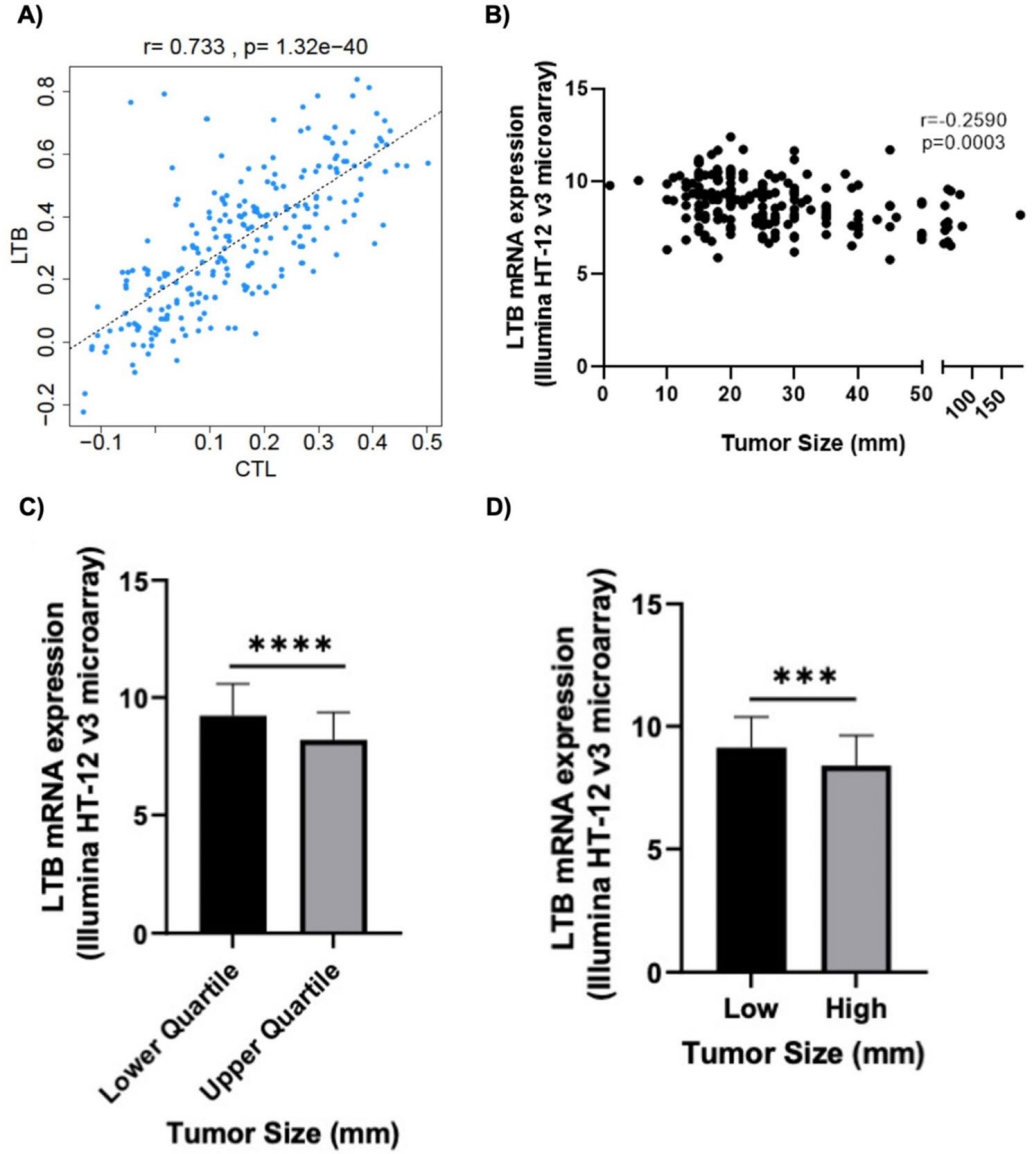
*Ltb* is associated with increased cytotoxic levels and decreased tumor size. (**A**) Correlation between *Ltb* and CTL levels from the TCGA METABRIC TNBC cohort analyzed by the TIDE query gene function. (**B**) Correlation between *Ltb* (mRNA expression, Illumina HT-12 V3 microarray) and tumor size (mm) (*n* = 187). *Ltb* expression stratified by (**C**) quartile (*n* = 56 lower quartile, *n* = 47, upper quartile) and (**D**) median tumor size (*n* = 107 low, *n* = 80 high). All TNBC subtype data abstracted from the TCGA METABRIC Nature 2012 & Nat Commun 2016 cohort. ***p < 0.0005. ****p < 0.00005, as indicated. Error bars denote standard deviation

CTL, cytotoxic lymphocytes; TCGA, The Cancer Genome Atlas; TNBC, triple negative breast cancer; TIDE, Tumor Immune Dysfunction and Exclusion; RFS, relapse free survival; OS, overall survival.

To investigate the utility of *Ltb* as a clinical prognostic biomarker, we analyzed publicly available TNBC subtype data obtained from TCGA Breast METABRIC study. TIDE analysis revealed that *Ltb* positively correlated with CTL levels (*r* = 0.733, *p* < 0.0001) (Fig. 5A) and negatively correlated with tumor size (*r*=-2590, *p* = 0.0003) (Fig. 5B). *Ltb* expression was also significantly (*p* < 0.0001) higher in smaller sized tumors (Fig. 5C-D). Interestingly, *PD-1* and *NFKB1* also demonstrated significant negative correlations with tumor size, however, the strength of these two correlations was weaker compared *Ltb* (Supplementary Table 1).

**Fig. 6:**
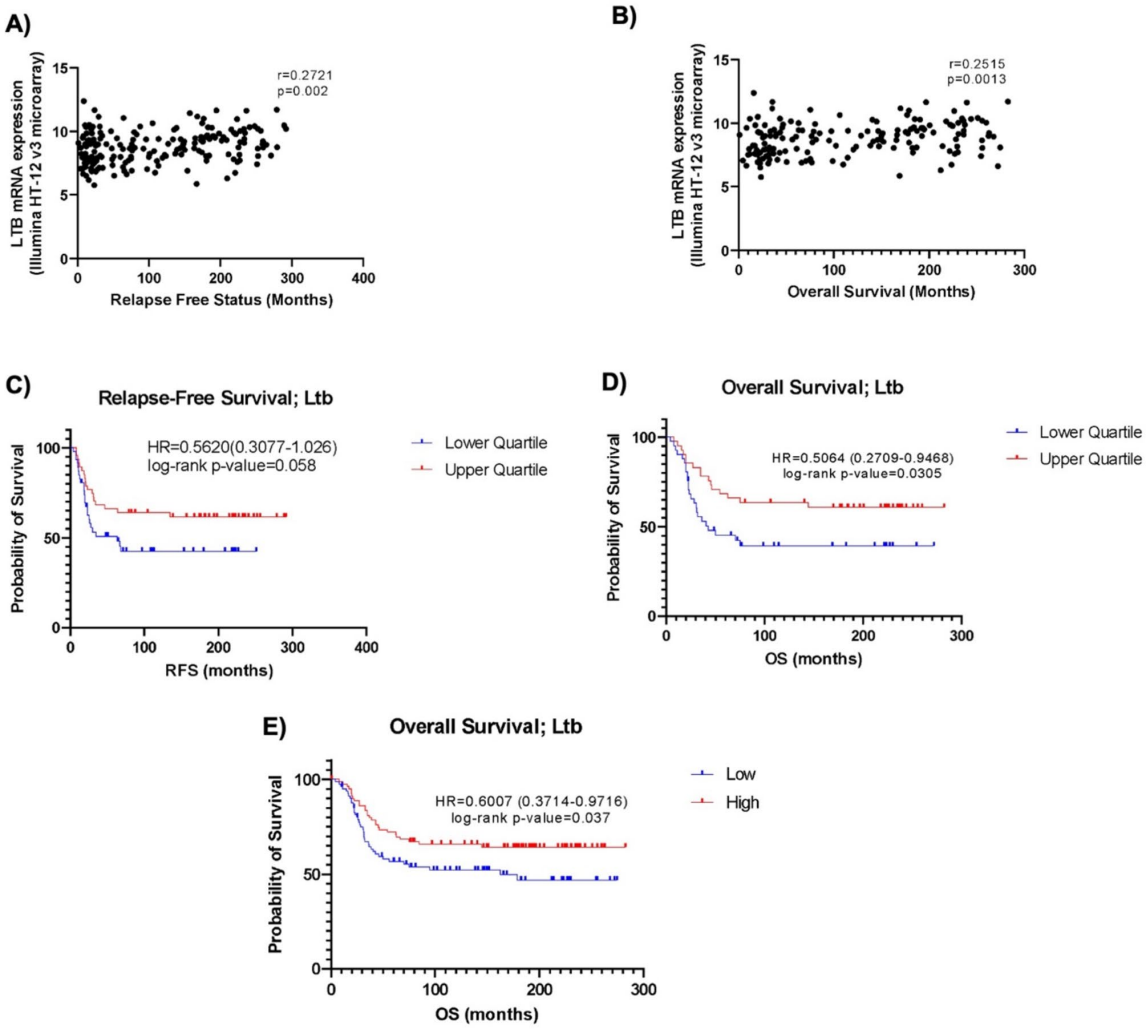
*Ltb* is associated with improved survival outcomes in TCGA TNBC METABRIC cohort. Correlation analysis between *Ltb* and (**A**) RFS (*n* = 187) and (**B**) OS (*n* = 161). Kaplan Meier curve analysis of *Ltb*’s association with (**C**) RFS stratified by lower and upper quartile *Ltb* expression and OS stratified by (**D**) lower and upper quartile and (**E**) median *Ltb* expression. All TNBC subtype data abstracted from the TCGA METABRIC Nature 2012 & Nat Commun 2016 cohort. TCGA, The Cancer Genome Atlas; TNBC, triple negative breast cancer; RFS, relapse free survival; OS, overall survival

Next, TCGA data was employed to determine if *Ltb* was associated with improved survival outcomes in TNBC. It was found that *Ltb* was significantly positively correlated with longer relapse free survival (RFS) (*r* = 0.2721, *p* = 0.002) and OS (*r* = 0.2515, *p* = 0.0013) (Fig. 6A-B). While numerous other factors significantly positively correlated with RFS or OS (*PD-L1*, *PD-1*, *INFG*, *NFKB1)*, *Ltb* demonstrated the strongest correlation overall (Supplementary Tables 2–3). Furthermore, Kaplan Meier curve analysis revealed that upon stratification of *Ltb* expression into upper and lower quartiles, there was a narrowly insignificant (HR = 0.5620, *p* = 0.058) relationship detected between higher *Ltb* levels and improved RFS (Fig. 6C). However, a significant relationship between higher *Ltb* levels and longer OS was observed when stratifying by both quartile and median expression levels (*p* < 0.05) (HR = 0.5064, *p* = 0.0305) (HR = 0.6007, *p* = 0.037) (Fig. 6D-E).

**Fig. 7:**
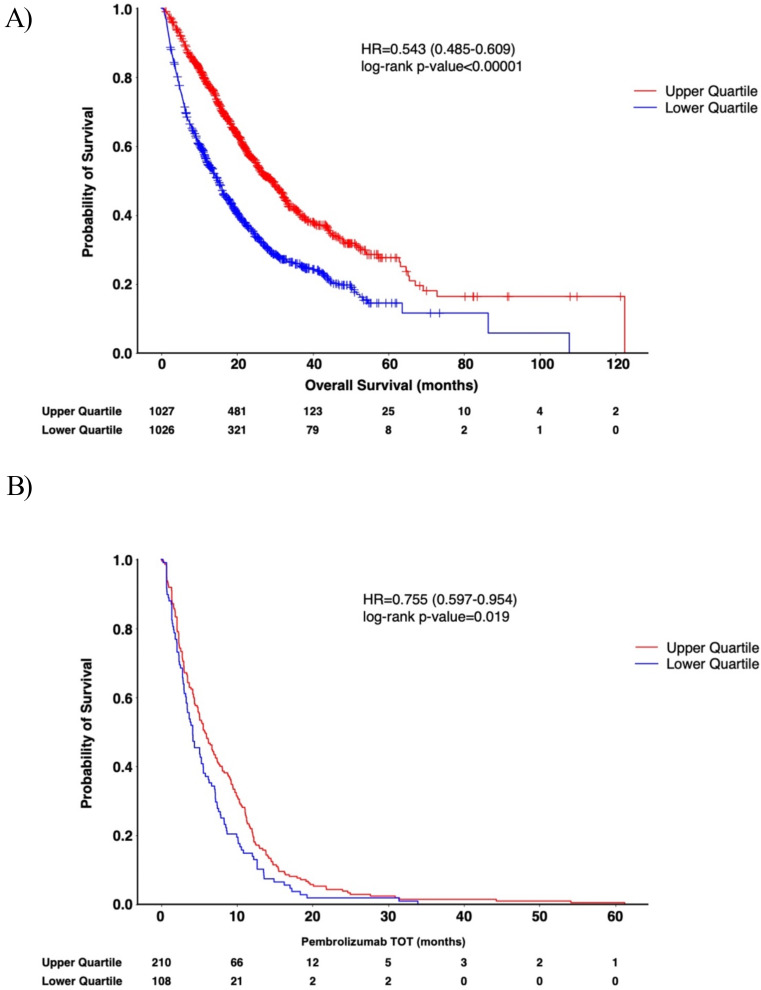
*Ltb* is associated with improved survival outcomes in TNBC patients treated with anti-PD-1 therapy. Kaplan Meier curve analysis revealed upon stratification of *Ltb* by upper and lower quartile that higher *Ltb* expression was significantly associated with improved (**A**) OS in TNBC patients and (**B**) TOT in pembrolizumab-treated TNBC patients (*n* = 1038 patients each in top and bottom quartile). TNBC, triple negative breast cancer; TOT, time on treatment; OS, overall survival

Finally, since patients in the TCGA TNBC cohort were not exposed to PD-1 based therapy, we examined a commercial database of real-world patient samples with matched insurance claims data to examine the prognostic value of *Ltb*. Kaplan-Meier curve analysis of TNBC patients demonstrated a significantly longer OS in patients with higher *Ltb* expression (Fig. 7A) (HR = 0.543, *p* < 0.00001), corroborating our findings in the TCGA cohort. In addition, pembrolizumab-treated TNBC patients with higher *Ltb* expression exhibited a significantly improved time on treatment (TOT) compared to patients with lower *Ltb* expression (HR = 0.755, *p* = 0.019) (Fig. 7B). Collectively, these findings demonstrate that *Ltb* is highly prognostic for improved TNBC patient survival outcomes, as evidenced by its association with prolonged survival in a real-world cohort of pembrolizumab treatment, which includes metastatic TNBC tumors.

### Standard-of-care combinatorial treatment reduces tumor burden in E0771 model, no effect in 4T1 model in vivo

**Fig. 8:**
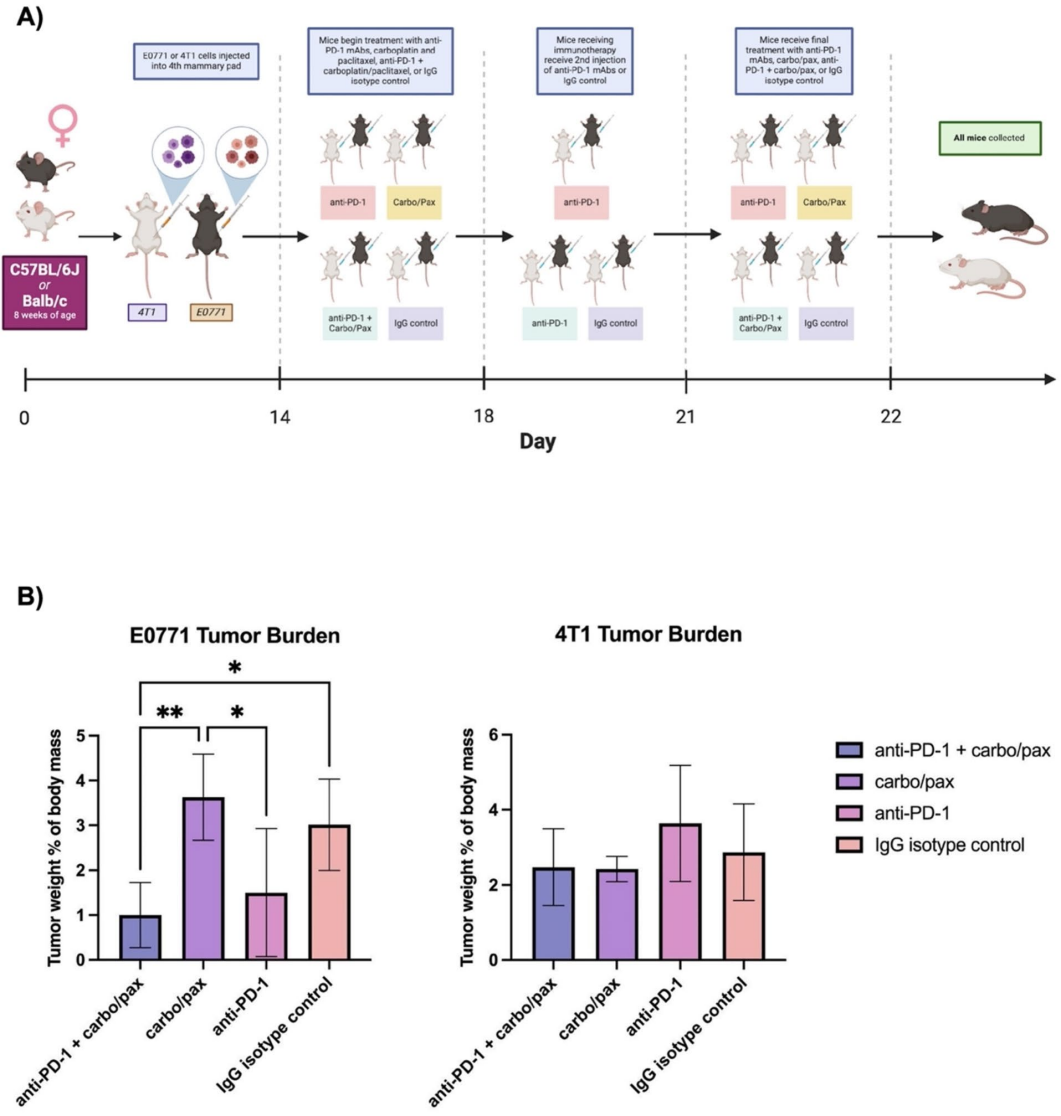
E0771 and 4T1 TNBC standard-of-care frontline treatment regimen in vivo study. (**A**) Experimental timeline. Created with BioRender.com. (**B**) Tumor burden analysis following combinatorial PD-1 inhibition and chemotherapy (carboplatin and paclitaxel) treated mice in the E0771 and 4T1 models (*n* = 5 per group). Error bars denote standard deviation. *p < 0.05, **p < 0.01, as indicated

To further expand the clinical relevance of our previous in vivo studies, we analyzed the effect of the frontline chemotherapies carboplatin and paclitaxel (carbo/pax) in combination with anti-PD-1 therapy in two different mouse models. We employed a 4T1 model to reflect a “cold” tumor microenvironment that is a characteristic of late-stage or metastatic TNBC to complement the E0771 model, which is representative of an early stage “hot” tumor microenvironment [42]. Indeed, previous studies have found that the E0771 model is responsive to immune checkpoint blockade, while the 4T1 model has been reported to demonstrate more dampened and variable responses to immunotherapy [43]. Prior to initiating studies, we evaluated PD-L1 levels via western blot in both E0771 and 4T1 cells and found that PD-L1 expression was decreased in the 4T1 model (Supplementary Fig. 4). A full study schema can be seen in (Fig. 8A). In the E0771 cohort, mice receiving the combination treatment of anti-PD-1 and carbo/pax had the lowest tumor burden, with significant decreases compared to the carbo/pax (*p* = 0.0062) and IgG isotype control groups (*p* = 0.0386) (Fig. 8B). Treatment with anti-PD-1 alone also significantly reduced tumor burden compared to carbo/pax (*p* = 0.0277), though not as dramatically as the combination treatment. No significant differences in tumor burden were found between treatment groups in the 4T1 cohort, suggesting that this model is minimally responsive to the treatments administered (Fig. 8B). Indeed, when considering all mice that received anti-PD-1 in combination with carbo/pax, the E0771 model exhibited a significantly lower tumor burden than the 4T1 model (*p* = 0.0387). However, treatment with carbo/pax alone was more effective in controlling tumor growth in the 4T1 model than in the E0771 model (*p* = 0.0302). Among the groups that received anti-PD-1 monotherapy, E0771 mice had a lower mean tumor burden than the 4T1 mice, although this difference was narrowly insignificant (*p* = 0.0524). Due to the high degree of tumor regression elicited by anti-PD-1 therapy in the E0771 model, we would expect responses to be improved in a more immunogenic model.

### Analysis of serum cytokine profiles in E0771 and 4T1 in vivo models

**Fig. 9:**
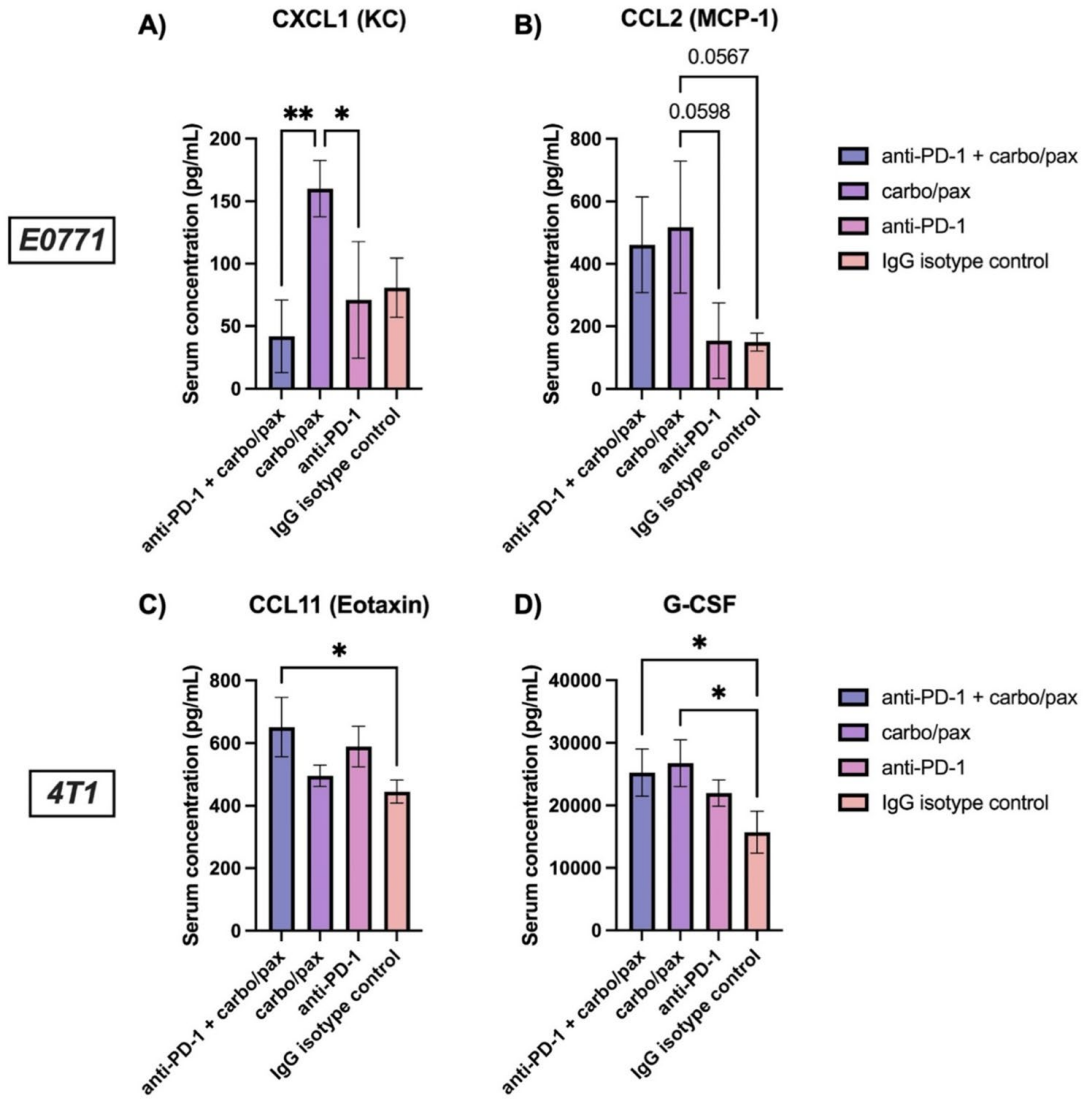
Serum cytokine analysis for E0771 and 4T1 TNBC standard-of-care frontline treatment regimen in vivo study. E0771 serum levels of (**A**) CXCL1 (KC) and (**B**) CCL2 (MCP-1). 4T1 serum levels of (**C**) CCL11 (Eotaxin) and (**D**) G-CSF. Error bars denote standard deviation. *p < 0.05, **p < 0.01, as indicated

Next, we again used a multiplex array to profile cytokines in serum from E0771 and 4T1 mice. Serum was collected from mice on the day following their final treatment with the goal of providing clinical utility for assessing therapeutic efficacy at an “on-treatment” timepoint, therefore potentially informing future treatment decisions. In the E0771 cohort, the only cytokine that exhibited significant differences between groups was CXCL1 (KC) (Fig. 9A). Interestingly, CXCL1 was significantly higher in the carbo/pax group when compared with mice receiving the combination treatment (*p* = 0.0084) or anti-PD-1 alone (*p* = 0.0374). This finding was somewhat surprising given that serum taken from the anti-PD-1 group two weeks following their final treatment had higher levels of CXCL1 compared to controls or other ICIs (Fig. 3I). However, these findings may indicate that CXCL1 exhibits temporal kinetics in response to PD-1 blockade. CCL2 (MCP-1) also showed a trend towards elevated levels in E0771 mice receiving carbo/pax treatment when compared to anti-PD-1-treated mice (*p* = 0.0598) and IgG controls (*p* = 0.0567) (Fig. 9B). In the 4T1 cohort, CCL11, a chemokine also known as Eotaxin that selectively recruits eosinophils and is associated with increased immune cell infiltration in breast cancer [44], was significantly higher in the combination treatment group than in IgG isotype controls (*p* = 0.0161) (Fig. 9C). In addition, G-CSF was elevated in the combination treatment (*p* = 0.0314) and carbo/pax (*p* = 0.0148) groups in comparison to IgG isotype controls (Fig. 9D). G-CSF is a proinflammatory cytokine that stimulates the production of myeloid immune cells and is commonly used as a treatment for chemotherapy-induced neutropenia [45]. However, its role in breast cancer is debated, as it has been shown to have pro-tumorigenic effects in the tumor microenvironment and found to be elevated in serum of breast cancer patients [45].

**Fig. 10:**
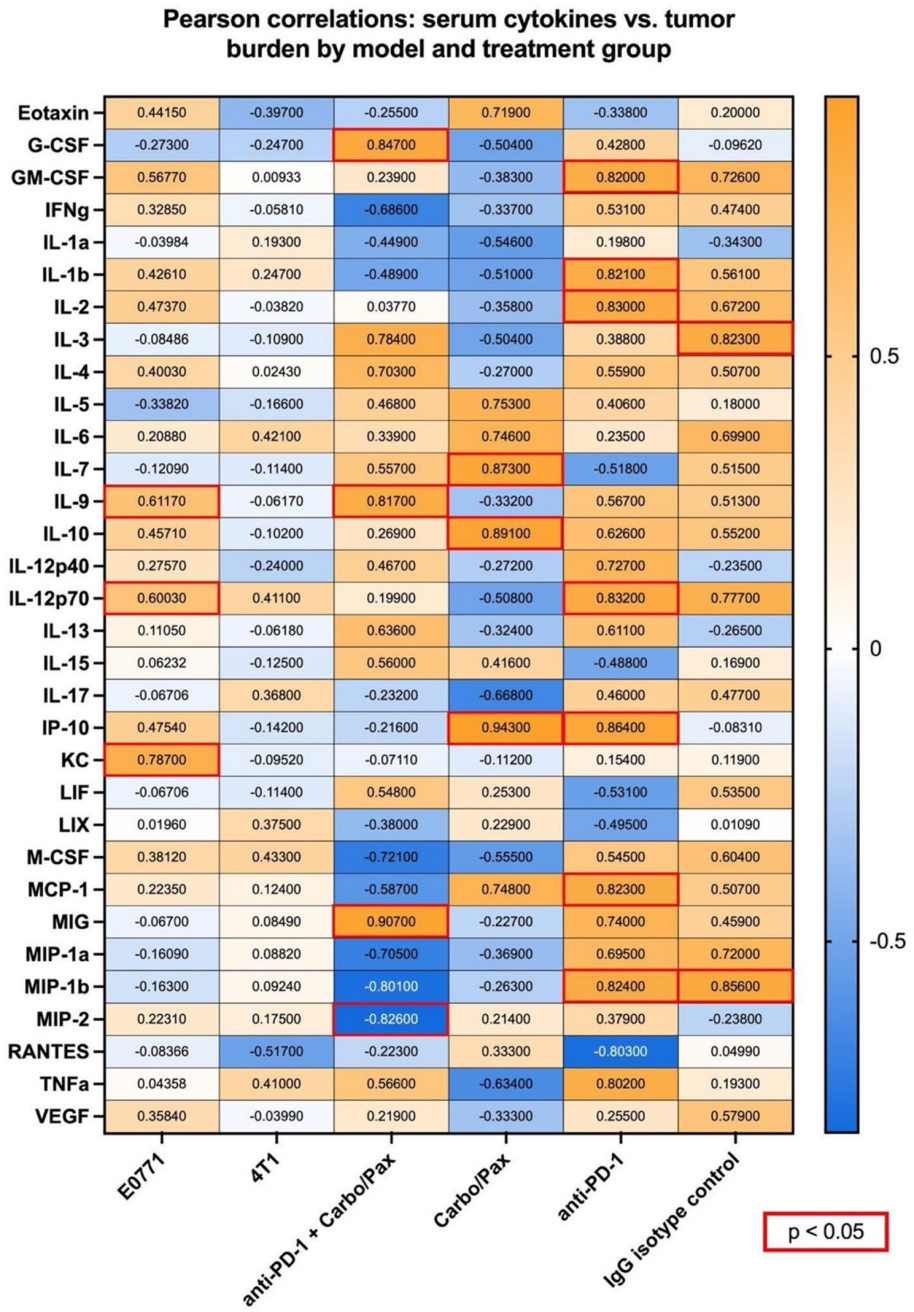
E0771 and 4T1 TNBC standard-of-care frontline treatment regimen. Pearson correlations of post-treatment serum cytokine levels with tumor burden, with statistically significant values outlined in red

We then aimed to assess model- and treatment-specific relationships between circulating cytokines and disease severity by determining the correlation of individual cytokines with tumor burden (Fig. 10). Similar to the aforementioned E0771 study, the cytokines that positively correlated with tumor burden in this E0771 cohort were IL-9 (*r* = 0.6117, *p* = 0.0346), IL-12p70 (*r* = 0.6003, *p* = 0.0390), and CXCL1 (KC) (*r* = 0.7870, *p* = 0.0024). IL-12p70, the active heterodimer form of IL-12, is a potent proinflammatory cytokine that is known to potentiate T and NK cell cytotoxicity in the tumor microenvironment [46]. Interestingly, there were no cytokines significantly correlated with tumor burden in the 4T1 cohort. We then performed comparisons based on treatment groups to evaluate treatment-specific effects in animals of both model cohorts. Within the combination treatment groups of anti-PD-1 and carbo/pax, the cytokines positively correlating with tumor burden were G-CSF (0.84700, *p* = 0.0334), IL-9 (*r* = 0.8174, *p* = 0.0470), and CXCL9, also known as MIG (*r* = 0.9072, *p* = 0.0125). Conversely, CXCL2, also known as MIP-2, showed a negative correlation with tumor burden (*r*=-0.8264, *p* = 0.426). CXCL9 is a pleiotropic chemokine that can have immunostimulatory effects within the tumor microenvironment but has been reported to be elevated in patients with ER-negative metastatic breast cancer [47]. Similarly, some studies have found CXCL2 to have a pro-tumorigenic role in breast cancer, but others have found that it may promote anti-tumor immunity by potentiating responses to PD-1 blockade and increasing the infiltration of anti-tumorigenic N1 neutrophils in TNBC [48]. In mice receiving only carbo/pax chemotherapy, strong positive correlations with tumor burden were found for IL-7 (*r* = 0.8734, *p* = 0.0230), IL-10 (*r* = 0.8912, *p* = 0.0171), and CXCL10 (IP-10) (*r* = 0.9433, *p* = 0.0047). The anti-PD-1-treated monotherapy groups had the highest number of positive correlations with tumor burden, with GM-CSF (*r* = 0.8201), IL-1β (*r* = 0.8214), IL-2 (*r* = 0.8297), IL-12p70 (*r* = 0.8318), CXCL10 (IP-10) (*r* = 0.8637), CCL2 (MCP-1) (*r* = 0.8231), and CCL4 (MIP-1β) (*r* = 0.8239) all reaching statistical significance (*p* < 0.05). Within IgG isotype controls, the cytokines with significant (*p* < 0.05) negative correlations were IL-3 (*r* = 0.8227) and CCL4 (MIP-1β) (*r* = 0.8562). There were no significant differences between treatment groups in intratumoral expression of *Ltb* and other NF-κB-related genes (Supplementary Fig. 5), however upon examining mean intensity and IOD levels of Ltb in PD-1 versus IgG control E0771 and 4T1 tumors by fluorescent immunohistochemistry we see that Ltb is significantly upregulated in E0771 PD-1 treated tumors (Supplementary Fig. 6). Overall, this discrepancy indicates that higher sample sizes may be required to adequately assess model- or treatment-specific effects on NF-κB signaling in the context of a clinically relevant dual chemotherapy and immunotherapy blockade.

## Discussion

The goal of this study was to identify novel immune markers of prognosis in TNBC, which still represents a critical unmet need. Currently, intratumoral PD-L1 expression remains the only clinically-approved marker to be used prospectively in ICI clinical trials for TNBC [16], which fails to recapitulate the high degree of tumor heterogeneity that TNBC patients exhibit. Other immune-based prognostic markers include microsatellite instability and high tumor mutational burden, but these are approved as tissue agnostic biomarkers and only represent a small proportion of TNBC cases [12]. Intratumoral and stromal TILs are still being evaluated for their prognostic utility, but challenges in standardization and reproducibility remain barriers to clinical integration [49]. Gene expression signatures and proteogenomic analyses are promising tools for disentangling issues presented by heterogeneity in TNBC, but require further investigation to validate their prognsotic value for therapeutic response [16, 50].

NF-κB signaling has the ability to induce PD-L1 expression in a multitude of different cancer subtypes, specifically through the production of IFNγ in T cells. In addition, tumor immune factors responsible for PD-L1 upregulation may represent more accurate immune-based prognostic markers in TNBC [51]. Interestingly, NF-κB has demonstrated prognostic value in cancer regardless of whether its signaling led to enhanced PD-L1 expression [52]. However, to the best of our knowledge, there have been no published reports that have examined if NF-κB activity is associated with improved TNBC patient outcomes in a population that was exposed to PD-1 based immunotherapy until now. In addition to increased intratumoral expression of *Ltb*, anti-PD-1 treatment elicited superior anti-tumor efficacy when compared to anti-LAG-3 and anti-TIM-3 therapy. We also established the prognostic utility of *Ltb*, in TNBC patient populations that both have and have not received pembrolizumab. Interestingly, a study by Marchetti et al.. similarly reported that *LTB* levels were significantly associated with improved prognostic outcomes in TNBC [53]. Ltb and familial cytokines are canonically known to participate in the homeostasis and development of secondary lymphoid organs in addition to promoting inflammation in both the contexts of innate and adaptive immunity during host defense [54]. While Ltb has been understudied in cancer, one study showed that it promotes chronic hepatitis in a transgenic mouse model, leading to a malignant phenotype resembling hepatocellular carcinoma (HCC) [55]. Echoing these findings, it has also been reported that Ltb is upregulated in HCC and corresponds with an increase in inflammatory cells [54]. Conversely, in breast cancer, Ltb has been found to be overexpressed in breast tumors that contain high levels of high endothelial venules (HEVs), which are responsible for the infiltration of lymphocytes into tumors [56]. Furthermore, it was discovered that Ltb was mainly produced by dendritic cells (DCs), and in turn, these DC clusters were significantly associated with the density of HEVs, T and B cell infiltration, and improved clinical outcomes [37, 56]. While this study was not specific to the TNBC subtype, it similarly shows that *Ltb* is associated with improved prognosis. Additionally, in concordance with its roles in lymphoid genesis, Ltb has been shown to promote the formation of DC-T cell microaggregates in oropharyngeal squamous cell carcinoma tumors, leading to higher rates of immune responsiveness and overall survival [37]. One limitation of this current study is while our Nanostring analysis demonstrated concordance with NF-κB signaling and enhanced cytotoxic levels, and that patient *Ltb* levels significantly correlated with intratumoral CTLs, we did not validate this phenomeneon at the protein level. Therefore, elucidating in a comprehensive manner what immune cell subtypes Ltb is associated with is an important future direction. Taken together, although further investigation is required to elucidate the mechanism by which LTB improves TNBC patient outcomes, our study provides a starting point for its use as a prognostic marker in TNBC.

The E0771 and 4T1 models are useful tools for studying the wide range of immune responses that may occur in the highly heterogeneous disease of TNBC. Numerous studies have demonstrated that the E0771 model is more immunogenic, which is consistent with our studies showing a reliably strong response to PD-1 blockade and robust intratumoral and circulating immune profiles associated with treatment [43]. TIM-3 blockade in E0771 mice also elicited a statistically significant reduction in tumor size at the post-treatment timepoint but showed decreased *Ltb* expression and NF-κB signaling when compared to the anti-PD-1-treated group. Our research also found that the efficacy of anti-PD-1 immunotherapy improved with the addition of carbo/pax. Consistent with results from the KEYNOTE studies [5, 57], treatment with standard-of-care chemotherapy alone was not as effective in controlling tumor growth compared to combinatorial treatment with a PD-1 inhibitor. These results suggest that E0771 is indeed a representative model for early-stage TNBC that is responsive to PD-1 blockade. In contrast, the 4T1 model derived little to no therapeutic benefit from any of the treatments administered. This was especially evident when comparing responses to the combinatorial treatment of anti-PD-1 and carbo/pax, where the E0771 group showed dramatic tumor regression, while the 4T1 exhibited almost no effect at all. These findings were not particularly surprising given that previous studies have characterized 4T1 as being less immunogenic and poorly responsive to immune checkpoint blockade. However, when comparing groups treated with carbo/pax chemotherapy alone, the 4T1 model showed a significantly stronger response than the E0771 model. One possible explanation for this difference could be attributed to chemotherapy-induced immunosuppression. The E0771 model, being more immunologically active, may have been more affected by the dampening of immune function than the 4T1 model, which had decreased levels of immune activity to begin with. Ultimately, further research will be necessary in order to understand the differences in chemotherapy response that was observed between the two models.

CXCL10 (IP-10) and CCL2 (MCP-1) were both found to have significant associations with higher tumor burden in the E0771 ICI monotherapy study and the combined E0771 and 4T1 groups treated with anti-PD-1. Previous studies have reported that 4T1 cells increase macrophage production of CCL2, CXCL1, and CXCL2 by secreting GM-CSF [58]. Indeed, we also found higher levels of GM-CSF to be associated with higher tumor burden in the anti-PD-1-treated mice from the E0771/4T1 study, pointing to a possible pro-tumorigenic GM-CSF-dependent cascade driven by the 4T1 cohort. In addition to its immunosuppressive effects, IP-10 is also hypothesized to promote tumor cell proliferation in breast cancer, which may contribute to its strong association with tumor burden among the different models and treatment groups [59]. One particular cytokine that we observed differential effects in was IL-9, which was elevated in serum of the anti-PD-1-treated group and associated with decreased tumor burden in E0771 ICI monotherapy study. However, it was strongly correlated with increased tumor burden in the subsequent study among the E0771 cohort and in mice from both models who received the carbo/pax and anti-PD-1 combination treatment. A possible explanation for this disparity could be attributed to the difference in time elapsed between treatment and collection serum, wherein a number of biological mechanisms could be influencing serum cytokine kinetics. For example, compensatory mechanisms may contribute to higher levels of pro-tumorigenic cytokines at an earlier timepoint, before being overcome by therapeutic activity and abating at a later timepoint. In addition, these differences may be related to the presence of enhanced PD-1-mediated tumor regression at the post-treatment timepoint compared with on-treatment. Overall, it will be pertinent to examine both on- and post-treatment circulating prognostic markers in future prospective TNBC patient cohort studies.

In conclusion, future studies stemming from this investigation involve the development of a multidimensional immune prognostic marker in a prospective cohort of TNBC patient tumors and serum, which will allow for potential additional TNBC prognostic markers to be identified in a non-bias manner. Moreover, it will also be pertinent to evaluate the prognostic role of circulating Ltb, as a non-invasive marker would represent superior clinical utility. Overall, our study underscores the importance of developing studies to better understand varying immune prognostic signatures, with the ultimate goal of improving patient clinical outcomes and providing personalized treatment strategies for patients with TNBC.

## Electronic supplementary material

Below is the link to the electronic supplementary material.

Supplementary Material 1

Supplementary Material 2

Supplementary Material 3

Supplementary Material 4

Supplementary Material 5

Supplementary Material 6

Supplementary Material 7

Supplementary Material 8

## Data Availability

NanoString raw data files are accessible through GEO series accession number GSE279896. All other data is located within the manuscript or associated supplemental files.
