# Supplementary material for: NF-κB associated markers of prognosis in early and metastatic triple negative breast cancer
Source: Breast Cancer Res. 2024 Dec 2;26:175. doi: 10.1186/s13058-024-01925-3 (PMC11613493; doi:10.1186/s13058-024-01925-3)
Supplement: Supplementary file 1 — Supplementary Material 1 [file 13058_2024_1925_MOESM1_ESM.docx]

*Supplementary Figure Legends*

**Supplementary Figure 1.** Uncropped tumor western blots

**Supplementary Figure 2.** Tumor burden at the post- treatment timepoint (n=3 per treatment group). *p<0.05, as indicated.

**Supplementary Figure** **3**. NanoString pathway and cell type scores by treatment group.

**Supplementary Figure 4.** E0771 and 4T1 cell line protein analysis. Western blot analysis of LTBr, PD-L1, p105/p50, and p100/p52 with corresponding GAPDH loading control.

**Supplementary Figure 5.** qPCR analysis of E0771 and 4T1 tumors. *IFNg*, *Ltb*, *NfkB1*, and *NfkB2* levels in E0771 and 4T1 anti-PD-1 + carbo/pax, carbo/pax, anti-PD-1, and IgG isotype control treated tumors. Ns, non-significant

**Supplementary Figure 6.** Ltb fluorescent immunohistochemistry analysis. Representative images of Ltb expression in E0771 and 4T1 IgG control and PD-1 mab. treated tumors, along with calculated mean intensity and integrated optical density Ltb levels.

**Supplementary Table 1.** Tumor size correlations with immune markers of interest.

**Supplementary Table 2.** Relapse free survival (RFS) correlations with immune markers of interest.

**Supplementary Table 3.** Overall survival (OS) correlations with immune markers of interest.
