# Supplementary figures and images for: NF-κB associated markers of prognosis in early and metastatic triple negative breast cancer
Source: Breast Cancer Res. 2024 Dec 2;26:175. doi: 10.1186/s13058-024-01925-3 (PMC11613493; doi:10.1186/s13058-024-01925-3)

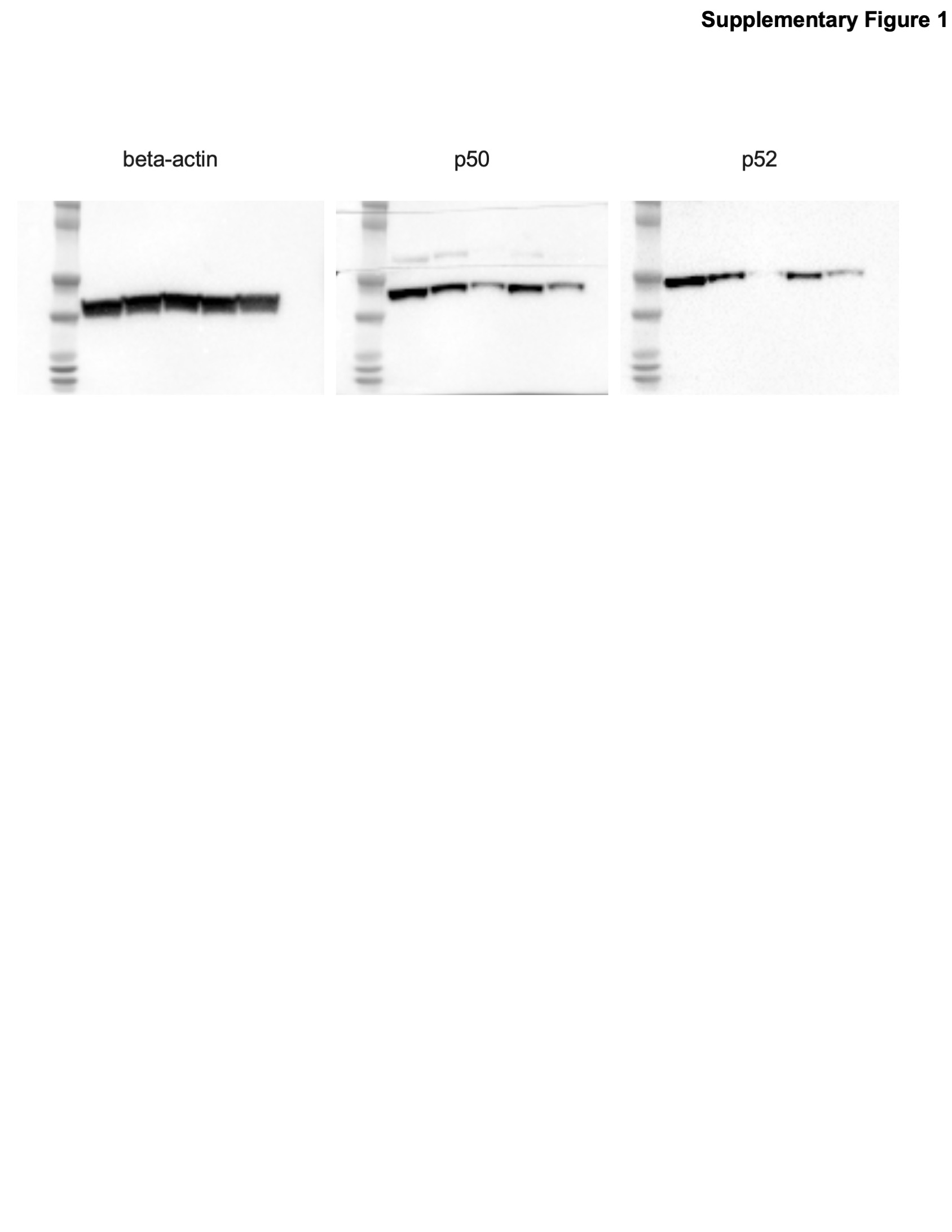

Supplement: Supplementary file 2 — Supplementary Material 2 [file 13058_2024_1925_MOESM2_ESM.jpeg]

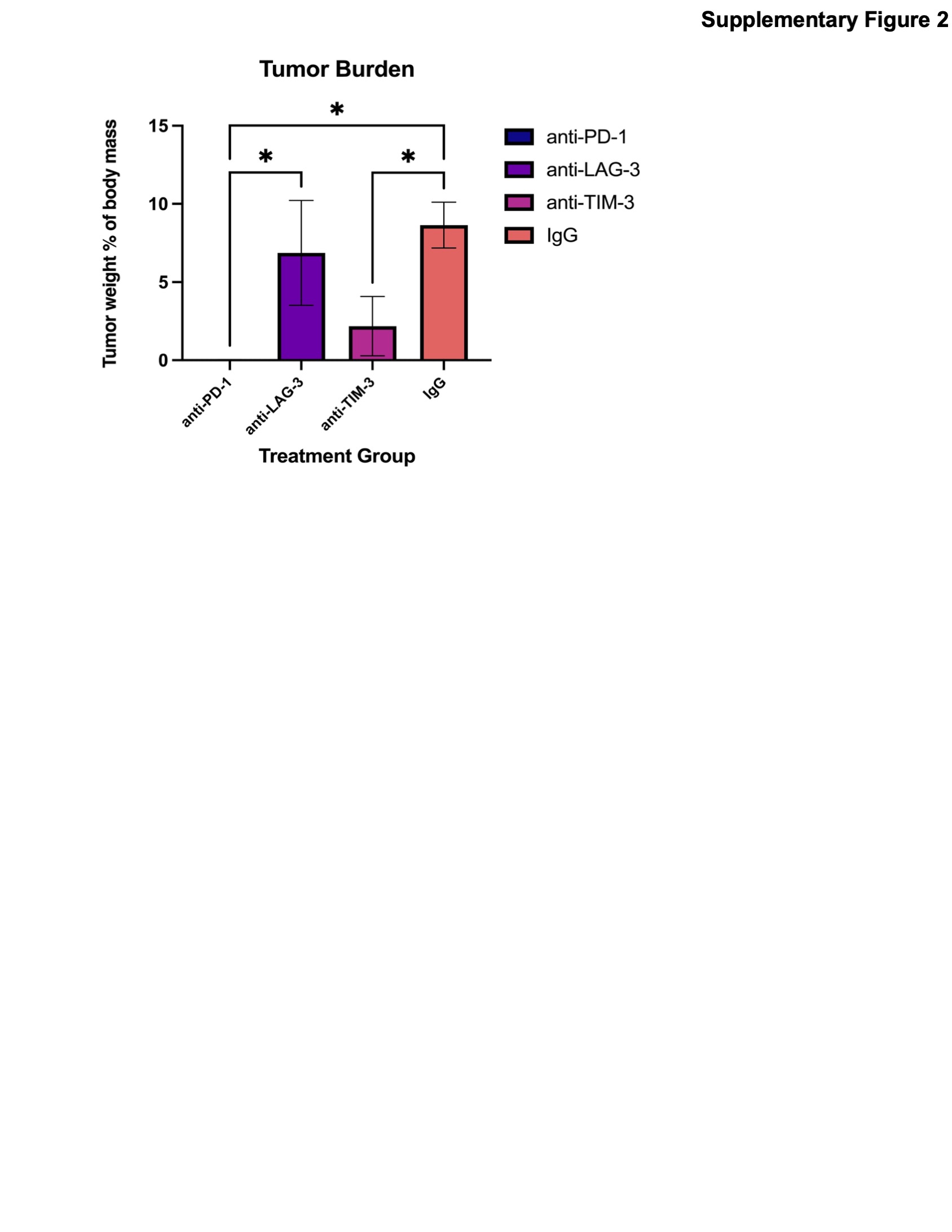

Supplement: Supplementary file 3 — Supplementary Material 3 [file 13058_2024_1925_MOESM3_ESM.jpeg]

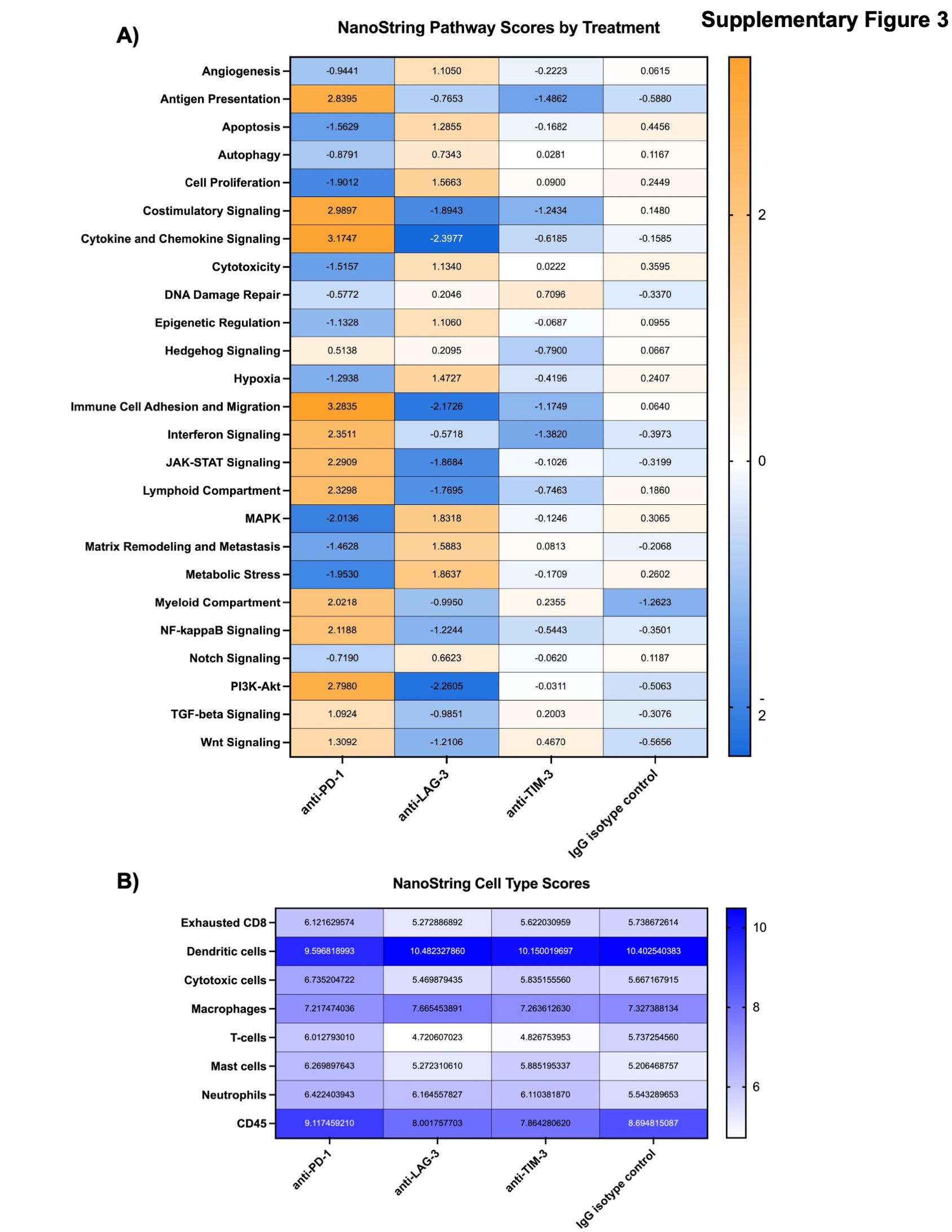

Supplement: Supplementary file 4 — Supplementary Material 4 [file 13058_2024_1925_MOESM4_ESM.jpeg]

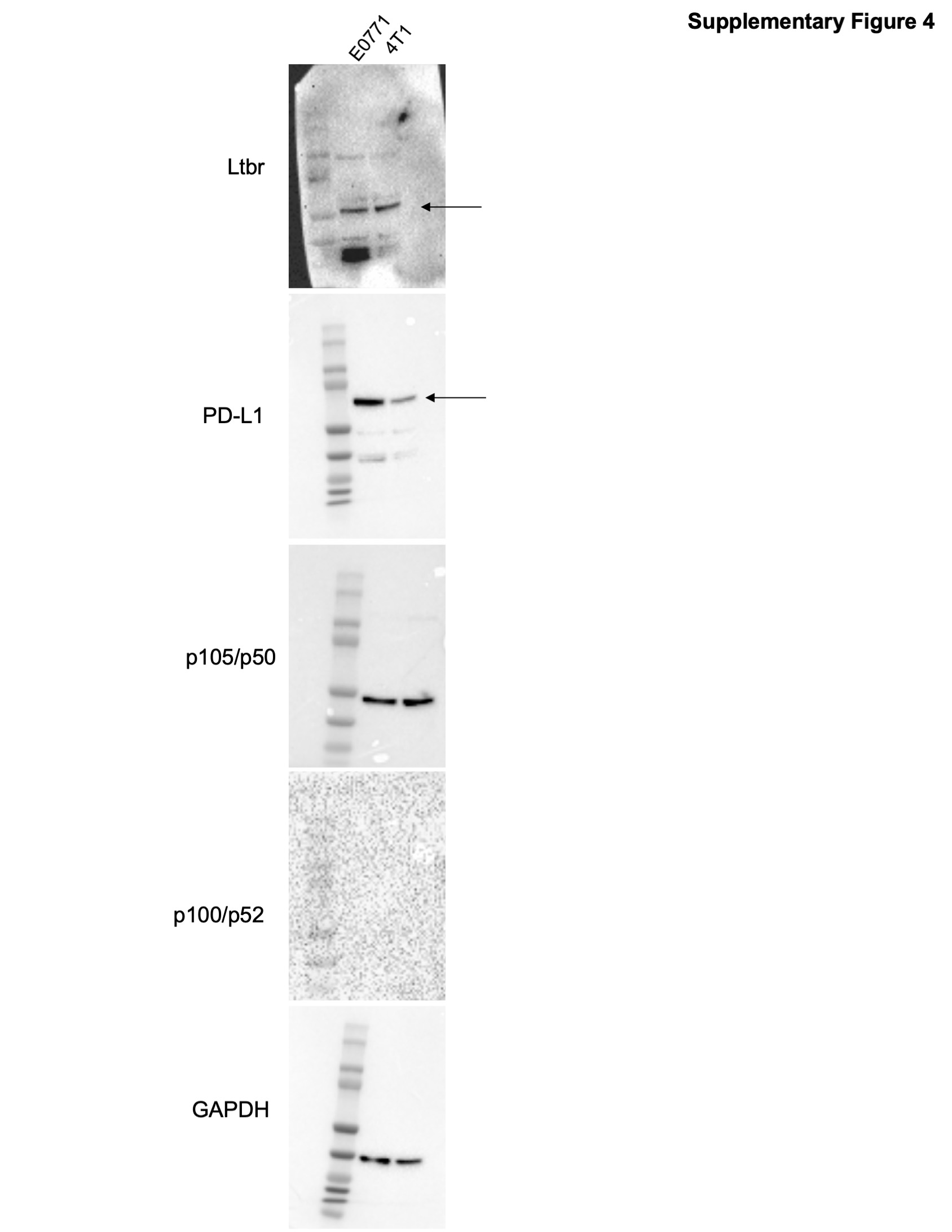

Supplement: Supplementary file 5 — Supplementary Material 5 [file 13058_2024_1925_MOESM5_ESM.jpeg]

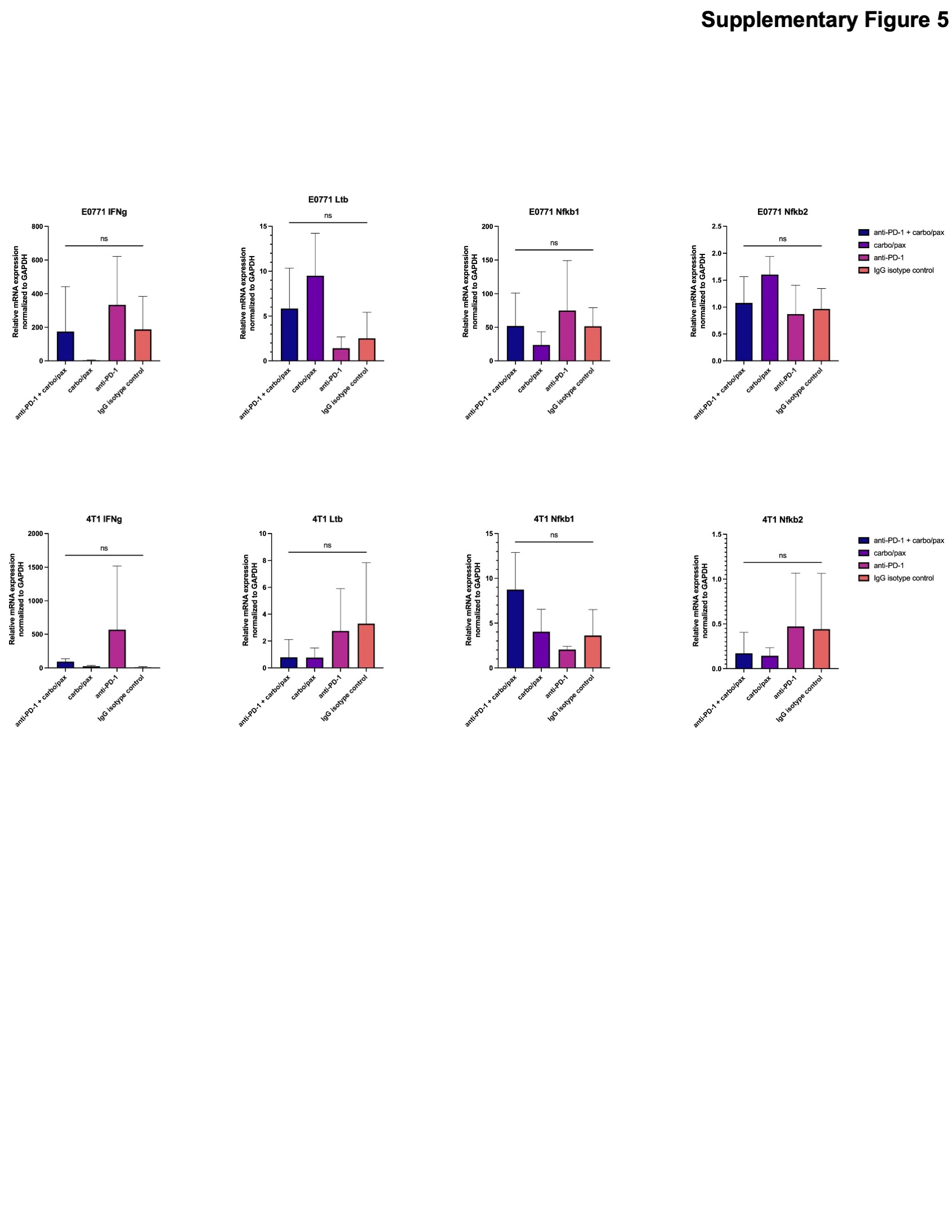

Supplement: Supplementary file 6 — Supplementary Material 6 [file 13058_2024_1925_MOESM6_ESM.jpeg]

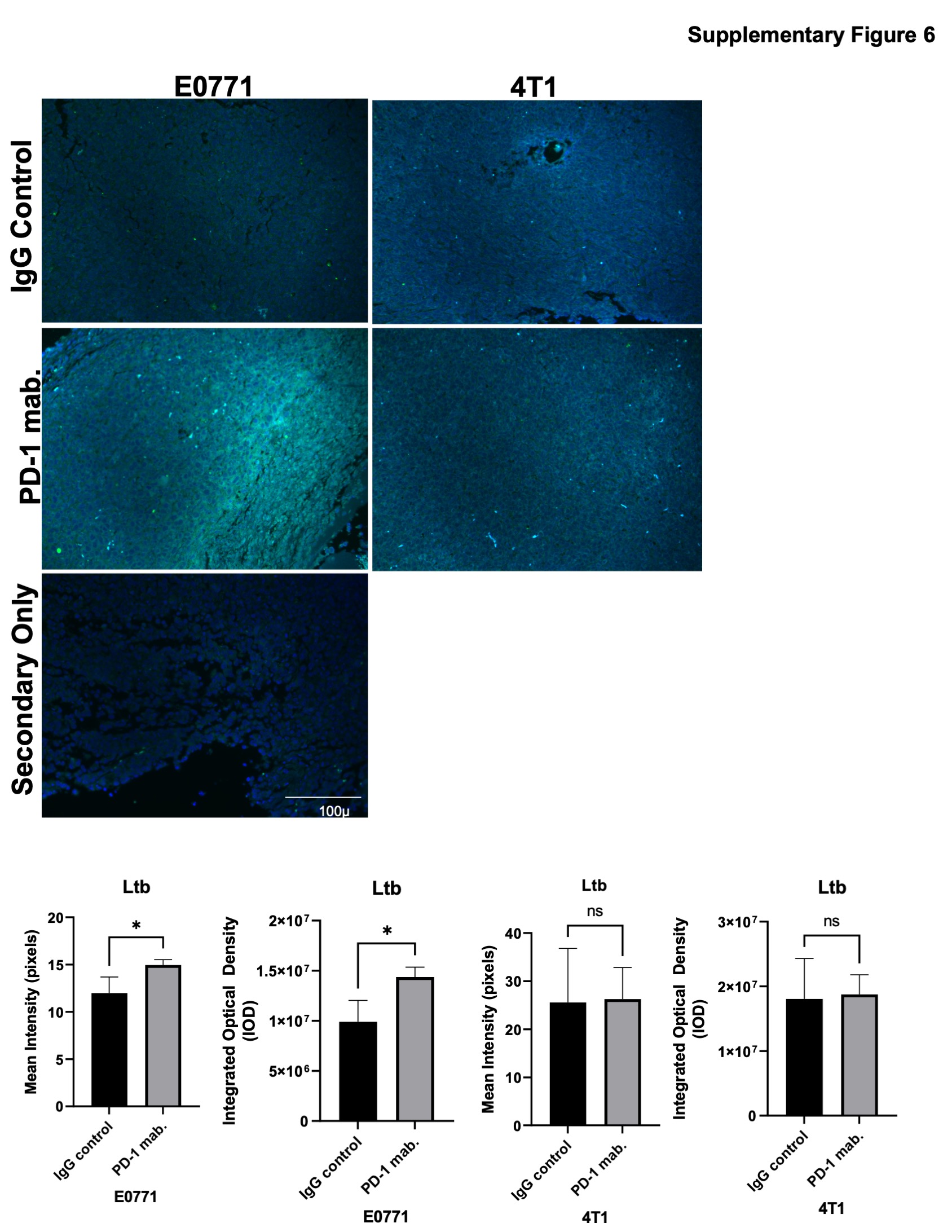

Supplement: Supplementary file 7 — Supplementary Material 7 [file 13058_2024_1925_MOESM7_ESM.jpeg]
