# Supplementary material for: NF-κB associated markers of prognosis in early and metastatic triple negative breast cancer
Source: Breast Cancer Res. 2024 Dec 2;26:175. doi: 10.1186/s13058-024-01925-3 (PMC11613493; doi:10.1186/s13058-024-01925-3)
Supplement: Supplementary file 8 — Supplementary Material 8 [file 13058_2024_1925_MOESM8_ESM.docx]

Supplemental Table 1 : Tumor Size Correlations with Immune Markers of Interest :

|  | R-value | p-value |
| --- | --- | --- |
| *LTBR* | 0.05489 | 0.4556 |
| *Ltb* | -0.2590 | 0.0003 |
| *PD-1* | -0.1579 | 0.0309 |
| *PD-L1* | 0.04453 | 0.5451 |
| *IFNG* | -0.1275 | 0.0821 |
| *NFKB1* | -0.1493 | 0.0414 |
| *NFKB2* | -0.02461 | 0.7381 |

Supplemental Table 2 : Relapse Free Survival (RFS) Correlations with Immune Markers of Interest :

|  | R-value | p-value |
| --- | --- | --- |
| *LTBR* | -0.06092 | 0.4076 |
| *Ltb* | 0.2721 | 0.0002 |
| *PD-1* | 0.2223 | 0.0022 |
| *PD-L1* | 0.1551 | 0.0341 |
| *IFNG* | 0.2366 | 0.0011 |
| *NFKB1* | 0.1459 | 0.0462 |
| *NFKB2* | 0.07366 | 0.3164 |

Supplemental Table 3 : Overall Survival (OS) Correlations with Immune Markers of Interest :

|  | R-value | p-value |
| --- | --- | --- |
| *LTBR* | -0.05242 | 0.5090 |
| *Ltb* | 0.2515 | 0.0013 |
| *PD-1* | 0.1473 | 0.0623 |
| *PD-L1* | 0.1599 | 0.0427 |
| *IFNG* | 0.1778 | 0.0240 |
| *NFKB1* | 0.1247 | 0.1150 |
| *NFKB2* | 0.02209 | 0.7809 |
